# GSC: efficient lossless compression of VCF files with fast query

**DOI:** 10.1093/gigascience/giae046

**Published:** 2024-07-19

**Authors:** Xiaolong Luo, Yuxin Chen, Ling Liu, Lulu Ding, Yuxiang Li, Shengkang Li, Yong Zhang, Zexuan Zhu

**Affiliations:** College of Computer Science and Software Engineering, Shenzhen University, Shenzhen 518060, China; BGI Research, Wuhan 430074, China; BGI Research, Shenzhen 518083, China; Guangdong Bigdata Engineering Technology Research Center for Life Sciences, BGI Research, Shenzhen 518083, China; Guangzhou Institute of Technology, Xidian University, Guangzhou 510555, China; National Engineering Laboratory for Big Data System Computing Technology, Shenzhen University, Shenzhen 518060, China; BGI Research, Wuhan 430074, China; BGI Research, Shenzhen 518083, China; Guangdong Bigdata Engineering Technology Research Center for Life Sciences, BGI Research, Shenzhen 518083, China; BGI Research, Wuhan 430074, China; BGI Research, Shenzhen 518083, China; Guangdong Bigdata Engineering Technology Research Center for Life Sciences, BGI Research, Shenzhen 518083, China; BGI Research, Wuhan 430074, China; BGI Research, Shenzhen 518083, China; Guangdong Bigdata Engineering Technology Research Center for Life Sciences, BGI Research, Shenzhen 518083, China; National Engineering Laboratory for Big Data System Computing Technology, Shenzhen University, Shenzhen 518060, China

**Keywords:** VCF/BCF files, lossless compression, rapid random access

## Abstract

**Background:**

With the rise of large-scale genome sequencing projects, genotyping of thousands of samples has produced immense variant call format (VCF) files. It is becoming increasingly challenging to store, transfer, and analyze these voluminous files. Compression methods have been used to tackle these issues, aiming for both high compression ratio and fast random access. However, existing methods have not yet achieved a satisfactory compromise between these 2 objectives.

**Findings:**

To address the aforementioned issue, we introduce GSC (Genotype Sparse Compression), a specialized and refined lossless compression tool for VCF files. In benchmark tests conducted across various open-source datasets, GSC showcased exceptional performance in genotype data compression. Compared with the industry’s most advanced tools (namely, GBC and GTC), GSC achieved compression ratios that were higher by 26.9% to 82.4% over GBC and GTC on the datasets, respectively. In lossless compression scenarios, GSC also demonstrated robust performance, with compression ratios 1.5× to 6.5× greater than general-purpose tools like gzip, zstd, and BCFtools—a mode not supported by either GBC or GTC. Achieving such high compression ratios did require some reasonable trade-offs, including longer decompression times, with GSC being 1.2× to 2× slower than GBC, yet 1.1× to 1.4× faster than GTC. Moreover, GSC maintained decompression query speeds that were equivalent to its competitors. In terms of RAM usage, GSC outperformed both counterparts. Overall, GSC’s comprehensive performance surpasses that of the most advanced technologies.

**Conclusion:**

GSC balances high compression ratios with rapid data access, enhancing genomic data management. It supports seamless PLINK binary format conversion, simplifying downstream analysis.

## Introduction

In recent decades, continuous advancements in technology and cost reductions in sequencing have resulted in a significant increase in large-scale sequencing projects, leading to a rapid growth of genotypic data. Currently, the variant call format (VCF) is the most commonly used format for storing DNA polymorphism data, encompassing single nucleotide polymorphisms, insertions, deletions, structural variations, and extensive annotations [1]. However, as a text-based format, VCF files occupy substantial storage space due to inherent redundancy. With the escalation of large-scale sequencing projects, the number and size of VCF files experience a dramatic surge. For example, the 1000 Genomes Project [2] and the analysis of whole-genome sequencing (WGS) of 150,119 individuals from the UK Biobank [3] generated VCF files in the hundreds of terabytes. Considering future projects that could scale to millions of samples, storing, transferring, and analyzing VCF files present increasingly challenging tasks. To address these issues, general-purpose compression methods, as well as more compact binary formats like BCF [4], have been widely employed. However, the compression ratios provided by these methods or formats are inadequate for handling large WGS genotype data contained in VCF files.

In recent years, numerous specialized compression algorithms for VCF files have emerged to improve the efficiency of storage, maintenance, and transmission. These algorithms can be broadly classified into 2 categories.

The first category primarily focuses on achieving high compression ratios, without significant consideration for the random accessibility of the compressed data. For instance, GTShark [5] and SAV [6] employ positional Burrows–Wheeler transform (PBWT) [7] to reposition the variant record data. This enables the identification of more data redundancy and facilitates more efficient genotype compression. VCFShark [8], an extension of GTShark, enhances the compression of the entire VCF file by incorporating special processing of the variant descriptive information. Genozip [9] also offers a lossless compression solution for VCF files, considering both genotype and additional annotation information. It also supports basic random access to the compressed data. Although these methods achieve superior compression ratios, they may not provide rapid random access to genotype data, which could be vital for subsequent analyses.

The second category involves the partitioning and reorganization of genotype data in order to achieve a balance between high compression ratios and efficient genotype retrieval. For instance, the aforementioned study introducing PBWT [7] also utilizes PBWT and run-length encoding techniques to greatly improve the compression ratio of genotype data. This approach also facilitates efficient matching of haplotypes in terms of time and space. GQT [10] optimizes the retrieval of individual genetic variations by transposing genotype data and applying word-aligned hybrid compressed bitmap indices. BGT [11] enables queries of genotypes and variants and efficiently manages complex variants in VCF files by separating sample phenotypes, site annotations, and genotypes and utilizing a 2-bit integer matrix combined with PBWT compression technology. GTRAC [12] achieves efficient compression of VCF files by building variant dictionaries and compressing binary matrices, and it provides specific query functionalities on the compressed data. SeqArray [13] offers users various efficient compression options and data access capabilities by utilizing the LZMA compression algorithm [14]. GTC [15] improves compression ratios and query speeds by rearranging genotype data in blocks and utilizing run-length and Huffman coding techniques. XSI [16] employs a hierarchical block compression strategy that leverages sparse coding, word-aligned hybrid encoding, and PBWT to achieve efficient genotype data compression. It uses BCF format to store the variant annotation information for random data retrieval. GBC [17] features partitioning and block segmentation, an efficient storage structure, and a parallel algorithm that significantly accelerates the query speed. GVC [18] achieves compression of gene sequence variations with random-access capability through the use of binarization, joint row- and column-wise sorting of variation blocks, and the efficient image compression codec JBIG [19]. Most of the above methods primarily focus on the compression of genotype data while disregarding the other annotation data present in VCF files. Given that genotype data might constitute only a portion of VCF files, such as in the first phase of the 1000 Genomes Project dataset  [2], relying solely on genotype compression is insufficient to alleviate the storage and transmission pressures of large VCF files.

In this article, we introduce GSC (Genotype Sparse Compression), a specialized and refined lossless compression tool designed for handling entire VCF files. GSC efficiently compresses both genotype data and annotation information within VCF files independently, enabling fast and diversified variant querying. It achieves exceptional compression ratios for both genotype data and the entire VCF file, while maintaining rapid data querying capabilities. Additionally, the compressed files generated by GSC can seamlessly be converted into the binary format required by PLINK, a widely used tool for genome-wide association studies [20]. This integration significantly accelerates downstream analysis. GSC offers a promising solution for storing VCF files by striking a fine balance between compression efficiency, random-access capability, and support for downstream analysis.

## Data Description

To evaluate the performance of GSC, we selected datasets from Phase 1 (1000GPip1: 1,092 samples, 39,707,426 variants) and Phase 3 (1000GPip3: 2,054 samples, 84,740,066 variants) of the 1000 Genome Project [21], as well as the dataset from sequencing project Mgp [22]. Each dataset comprises multiple VCF files, with each file containing data of a single chromosome. We merged all VCF files from the 1000GPip3 dataset into a single VCF file named Kgenome, specifically to evaluate whether the compressor can handle VCF files containing multiple chromosomes. More details of the datasets are provided in Section 2 of the Supplementary Data.

## Findings

### Compression performance

To evaluate the compression performance of GSC, we conducted a comparison between GSC and other representative state-of-the-art random-accessible VCF compressors, including GBC [17], GTC [15], XSI [16], and PBWT [7]. In addition, the general-purpose compressor gzip [23], zstd  [24], and BCFtools  [4] were also involved as the baselines. To ensure the fairness of the comparison, especially in the mode focusing solely on genotype data compression, we excluded all subdomains from the INFO and FORMAT data fields, except for the “GT” subdomain. GBC, GTC, and GSC all compress the data in a block-wise fashion. To avoid the potential biases introduced by using different block sizes, we configured the 3 methods with the same block size. All compressors were run with a single thread on the same operating system. The detailed software and hardware configurations are provided in Section 3 of the Supplementary Data.

**Figure 1:**
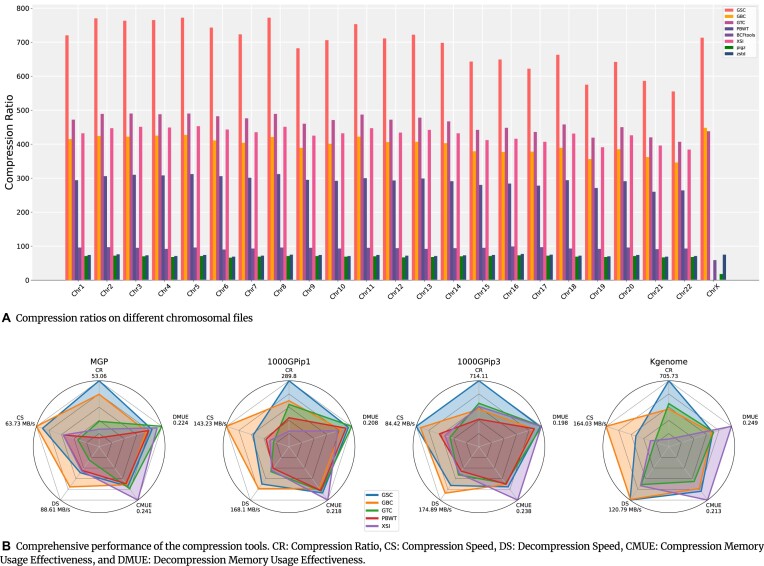
Compression results of genotype data. (A) Compression ratios of various tools in the dataset 1000GPip3 across 23 chromosomes files. (B) Comprehensive performance comparison of compressors on datasets Mgp, 1000GPip1, 1000GPip3, and Kgenome.

The compression ratios (original data size/compressed data size) of the compressors are summarized in Table  1, where the results demonstrate the superiority of GSC to the compared methods. GSC offers a highly competitive compression ratio in genotype data compression. For example, in the 1000GPip3 dataset that predominantly comprises genotype data, GSC achieves a compression ratio of 712.07, which is 1.5× to 5.5× of that of other random-accessible VCF compressors (i.e., GBC, GTC, XSI, and PBWT) and 8× to 10× of that of the general-purpose compressor gzip, zstd, and BCFtools. We also explored the efficiency of the compressors across different chromosomes within 1000Gpip3, as shown in Fig. 1A. GSC performs consistently across different chromosomes. PBWT and XSI failed to compress the dataset of the ChrX chromosome that contains genotypes of varying ploidy.

**Table 1:** Compression ratios of genotype data

	Variant	Original								
Datesets	sites	(GB)	gzip	BCFtools	zstd	PBWT	XSI	GTC	GBC	GSC
Mgp	90,310,977	18.57	23.68	25.79	17.10	7.2	14.09	20.41	42.2	**53.06**
1000GPip1	39,707,426	156.49	38.15	57.53	32.81	100.31	68.35	180.37	191.08	**289.80**
1000GPip3	84,740,066	794.36	63.37	88.60	72.53	172.77	435.29	465.92	391.54	**714.11**
Kgenome	84,740,066	785.10	62.32	91.21	71.61	—	—	461.71	392.55	**705.73**

“—” indicates the data cannot be successfully compressed by the corresponding method. Bold values indicate the best compression ratios attained in the corresponding datasets.

We further conducted an evaluation of the overall performance of the compared methods in terms of compression ratio (CR), compression speed (CS), decompression speed (DS), compression memory usage effectiveness (CMUE), and decompression memory usage effectiveness (DMUE). The memory usage effectiveness is defined as $1/\log _{10}^{T}$, where *T* is the peak size of memory (KB) used during compression/decompression. As shown in the radar chart in Fig. 1B, GSC demonstrates a good compromise performance over all 5 metrics, which could be estimated by the area covered on the chart, and superior in terms of compression ratio.

In addition to the genotype data compression, GSC also supports the compression of a whole VCF file. We compared GSC with the general-purpose compressors, including BCFtools, gzip, and zstd, and the specialized whole VCF file compressors (i.e., Genozip and VCFshark) in the compression of the whole VCF files in Table  2. Note that it might be inappropriate to directly compare GSC with fast random accessibility to the other methods that do not well support random access. Yet GSC still achieves significantly better compression ratios than the general-purpose compressors (i.e., BCFtools, gzip, and zstd). Since GSC has to maintain reasonable extra space to support the fast query functionality, its compression ratios are expectably inferior to VCFshark and Genozip, which are not intended to support fast random access. GBC, GTC, and PBWT were not included in this comparison since they cannot handle the whole VCF file.

**Table 2:** Compression ratios of the whole VCF file

Datasets	Variant sites	Original (GB)	gzip	BCFtools	zstd	Genozip	VCFshark	GSC*
Mgp	90,310,977	182.22	5.28	4.99	5.05	**9.17**	9.16	8.35
1000GPip1	39,707,426	878.37	8.08	6.13	6.05	17.48	**19.82**	17.32
1000GPip3	84,740,066	803.70	67.75	67.12	80.95	547.31	**563.12**	438.31
Kgenome	84,740,066	794.84	67.00	66.38	78.91	549.34	**557.54**	433.40

* GSC enable fast random access, whereas the other methods do not well support random access. Bold values indicate the best compression ratios attained in the corresponding datasets.

## Effects of the Key Components

GSC is featured by haplotype clustering and sparsification that leads to repositioning of the haplotypes. As described in the “Compression of genotype data” section, the new permutation order of the haplotypes denoted by an array *P* is the pivotal information that must be recorded to ensure lossless compression. To record *P*, in GSC, we reorder the POS (position) field according to *P* and store merely the reordered POS values (named as reordered mapping scheme). The original POS values and *P* can be fully recovered from the reordered POS values (as illustrated in Fig. 7). Alternatively, we can apply delta encoding to the POS values and store the array *P* as it is (named as direct storage scheme). To investigate the effectiveness of the reordered mapping scheme, we compared it with GSC using the direct storage scheme. The experimental results are reported in Table 3, where the reordered mapping scheme shows superior compression performance.

**Table 3:** Compression ratios: direct storage vs. reordered mapping

	GSC	GSC
	(direct	(reordered
Datesets	storage)	mapping)
MGP	46.89	**53.06**
1000GPip1	277.15	**289.80**
1000GPip3	698.99	**712.07**

Bold values indicate the better compression ratios attained in the corresponding datasets.

**Figure 2:**
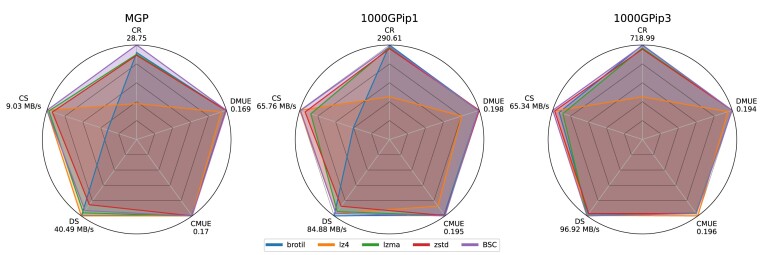
Performance comparison of using different backend compressors in GSC on datasets Mgp, 1000GPip1, and 1000GPip3.

As detailed in the “Methods” section, GSC uses BSC as the backend encoder for the compression of different data streams. The selection of backend encoder is critical to the overall performance of GSC. To investigate the effects of using different compressors as the backend, we evaluated GSC with mainstream compressors, including brotli  [25], zstd, lz4  [26], lzma  [27], and BSC. The comparison results in terms of compression ratio, compression and decompression speed, and memory use efficiency in 1000GPip1, 1000GPip3, and Mgp datasets are plotted in Fig. 2. brotli achieved superior compression ratios at the cost of compression speed. lz4 excelled in compression speed and memory efficiency but obtained lower compression ratios. lzma, zstd, and BSC attained better overall performance than brotli and lz4. Among them, BSC achieved the best compromise in terms of all performance metrics, which justifies our selection of BCS as the backend compressor in GSC.

### Query performance

Supporting rapid genotypic extraction is highly demanded in the compression of VCF files. GSC not only provides high compression ratios in both lossless and lossy compression modes but also enables swift and flexible genotypic querying, a feature not found in most generic compression tools. To evaluate the query performance of GSC, we selected the representative chromosome 1 from dataset 1000GPip3 as the target for genotype querying, which comprises 2,504 samples and 6,468,094 variants. GSC was compared with GBC, GTC, PBWT, BCFtools, XSI, and Genozip in both variant-based and sample-based querying. It is important to note that VCFshark was not included in this comparison as it does not support random access.

**Figure 3:**
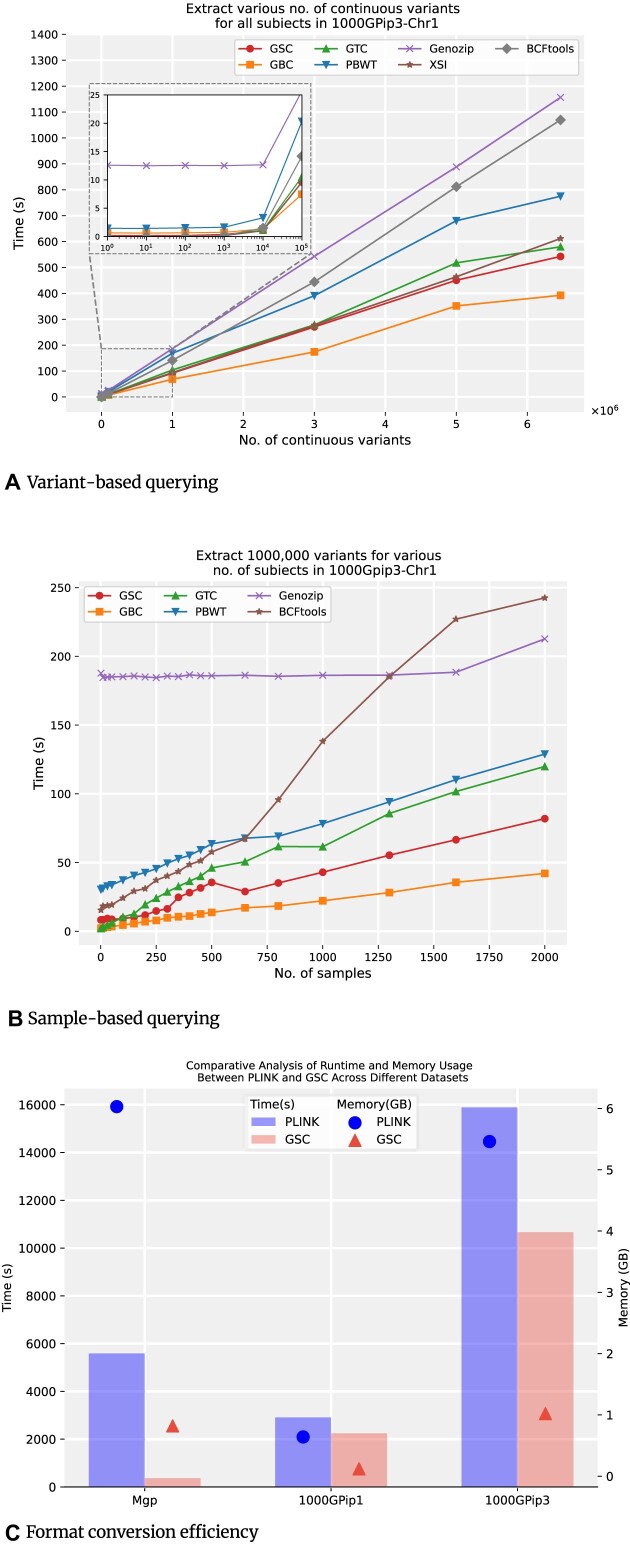
Query and format conversion performance. (A) Performance of querying various number of continuous variants for all samples in 1000GPip3-Chr1. (B) Performance of querying 1000,000 variants for various number of samples in 1000GPip3-Chr1. (C) Performance comparison between PLINK and GSC in conversion to PLINK binary format.

Query time for genotypes across various variant ranges using different tools is displayed in Fig. 3A. The sample size was consistently set to 2,504. In queries of less than 1,000 variant rows, GTC, XSI, and BCFtools completed them in just a few hundredths of a second, while GBC and GSC took slightly longer time (i.e., up to a tenth of a second). PBWT consumed 1 to 2 seconds to finish the same query. Genozip tended to be slower than other tools by taking up to 10 seconds. As the query range increased to over 10,000 variant rows, the querying speeds of GSC and GBC exceeded that of other methods.

The running times for querying different sample sizes of the methods within a set range of 1,000,000 variant rows are delivered in Fig. 3B. For queries involving fewer than 50 samples, GTC and GBC demonstrated comparable efficiency, with the query times ranging from 2 to 7 seconds. GSC, though marginally slower, completed queries for these smaller sample sets in approximately 8 seconds, while PBWT and BCFtools required substantially longer running time, often tens of seconds for equivalent tasks. As the sample count increased, GSC and GBC again showed an advantage against other tools. Genozip needed to decompress the entire file for queries, showing a consistent query time of around 190 seconds, regardless of the sample size. XSI performed comparably to GSC for single-sample queries but failed when querying multiple samples.

In summary, with larger query ranges, GBC consistently demonstrated the shortest query time, and GSC was the runner-up in both variant-based and sample-based querying. Genozip and BCFtools tended to be less efficient than other tools. For more details of the querying results, the reader is referred to Section 4 in the Supplementary Data.

### Efficiency of format conversion

To demonstrate the efficiency of format conversion from the output of GSC to PLINK binary format (i.e., “bed” format), we compared the conversion runtime and memory usage of GSC output to “bed” vs. VCF to “bed” using PLINK on Mgp, 1000GPip1, and 1000GPip3 datasets, as shown in Fig. 3C. Note that PLINK does not support direct conversion of VCF containing multiallelic genotypes to “bed.” Hence, BCFtools has to be used to preprocess the VCF files for PLINK. In contrast, GSC can efficiently handle multiallelic genotypes and variant description information during the VCF compression process (i.e., it enables direct conversion of the compressed file to “bed” format). As depicted in Fig. 3C, GSC consumes much less time and memory space to convert the file format. Particularly in smaller sample datasets like Mgp, GSC is 15 times faster than PLINK. In large datasets from the 1000 Genomes Project, GSC still manages to attain a speedup of 30% with much smaller memory usage. The considerable reduction in conversion time and memory consumption highlights the benefit of saving VCF data with GSC compression, especially for the scenarios where PLINK is a downstream analysis option.

## Discussion

In this article, by leveraging the sparse characteristics of preprocessed genotypes, we have crafted an efficient lossless compression algorithm namely GSC for VCF files, which supports fast genotype query. GSC attains competitive overall performance in terms of compression ratio, speed, memory usage, and query efficiency compared to other counterpart compressors. Specially, GSC shows superior compression ratios in both genotype compression and whole file compression. GSC also supports an efficient data conversion to PLINK binary format, which greatly facilitates the downstream analysis. For the sake of data management, GSC offers options to compress multiple VCF files into a single compressed archive (with the same sample count) and enables decompression of an archive into multiple VCF files according to the chromosomes. GSC also supports a streaming mode of operation that helps integrating GSC into pipelines. GSC can serve as a candidate-efficient solution for VCF files storage and management.

Despite the promising performance of GSC, there are still some limitations. For example, currently GSC cannot handle VCF datasets containing no genotype information. This limitation is inherent to the design of GSC, which features a high degree of coupling between genotype information and the POS field. It deserves future work to improve the generalization ability by exploring new efficient methodologies to handle such datasets. Moreover, the block size in genotype data compression is fixed to the number of samples, which could maximize the redundancy reduction in the haplotype clustering and sparsification. Yet exploring the configurations with different block sizes could provide further insights into the scalability of GSC and more flexibility to the user.

## Methods

**Figure 4:**
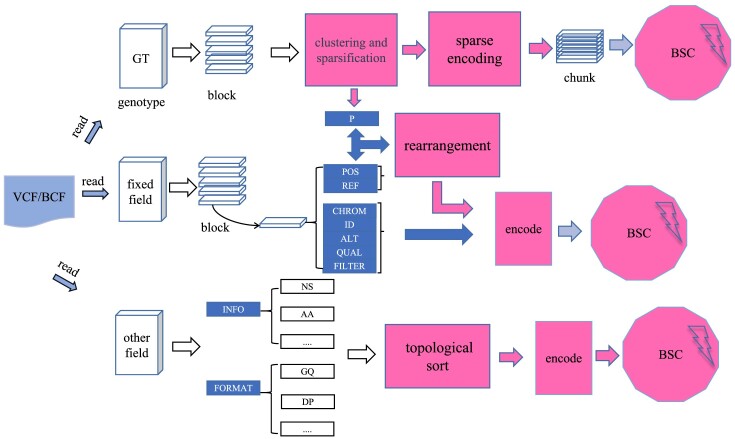
Overall workflow of GSC. In the initial step, VCF or BCF files are meticulously partitioned into multiple regions. Subsequently, differentiated processing strategies are applied based on the characteristics of each region to optimize the data structure. In the final step, all data, having been optimized, are further compressed using the BSC compressor to achieve efficient data storage.

The procedure of GSC is shown in a schematic diagram in Fig. 4. Given a VCF or BCF (binary version of VCF) file, GSC separates the annotation and genotype data and compresses them with different strategies. Particularly, GSC leverages a hierarchical and block-based compression strategy to compress the genotype data. The genotype data are first divided into blocks, each of which undergoes intrablock sorting, XOR processing, and sparse encoding. Afterward, the processed blocks are merged and encoded with general-purpose compressor BSC [28]. The data fields, including CHROM, POS, ID, REF, ALT, QUAL, and FILTER, are treated as fixed data streams. Each stream is partitioned into blocks of varying sizes, where each block’s data volume is decided by the number of variant points in a genotypic data block. The stream blocks are also compressed with BSC. The remaining INFO and FORMAT data fields may contain subfields. Each subfield (except the genotype) is divided into fixed-size blocks and compressed independently. GSC not only supports lossless compression of VCF files but also facilitates rapid querying of genotype data. The key components of GSC are detailed as follows.

### Preprocessing

**Figure 5:**
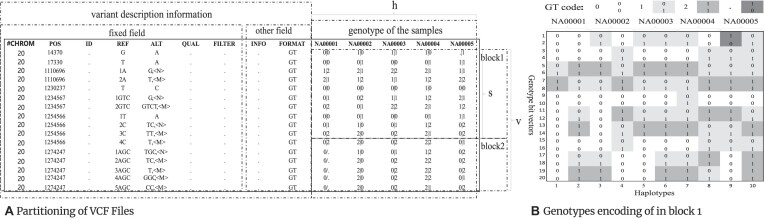
Preprocessing the input VCF file. (A) Splitting of specific variant rows and partitioning of VCF data for management. (B) Each genotype is encoded into 2 bits.

The input VCF/BCF file first undergoes preprocessing to conform with the following compression. As illustrated in Fig. 5A, an input VCF/BCF file is likely composed of data from *n* chromosomes with each possessing $v_i$ variants. Each variant, recorded in a line, contains *h* haplotypes denoted with “|” for phased and “/” for unphased alleles. Before compression, a multiallelic variant is converted to multiple distinct variants where the first alternative allele remains unchanged, while the subsequent ones are denoted by special markers. For instance, as shown in Fig. 5A, a variant at POS = 1110696 of alleles “G” and “T” is divided into 2 distinct variants: with the first marked as G, <N>, and the second as T, <M>, in ALT field. The <N> marker is exclusively used for the first split variant. To maintain the order of the variants, an additional index is added to the front of REF value for the variants with identical POS values. For example, indexes “1” and “2” are added in REF filed of the variants at POS = 1110696 as shown in Fig. 5A.

To encode the genotype data, each variant is represented by 2 variant bit vectors of size *h* following [15], as shown in Fig. 5B, where the bits indicate the type of mutation, that is, “00” for reference allele (“0”), “01” for nonreference allele (“1”), “11” for other nonreference alleles (“2”), and “10” for unknown alleles (“.”). As such, the genotype of each chromosome can be encoded with $2hv_i$ variant bits, and the genotype data of the entire VCF/BCF file are encoded with a total of $2h {\textstyle \sum _{i=1}^{n}}v_{i}$ variant bits.

### Compression of genotype data

After preprocessing, the bit vector–encoded genotype data are partitioned into blocks, with each block containing *s* consecutive variants (i.e., $2s$ variant bit vectors per block). If the number of haplotypes *h* is smaller than $2^{13}$, *s* is set to *h*; otherwise, *s* is set to $2^{13}$. Consequently, a complete block contains $2sh$ bits. A chromosome *i* is divided into $\left\lceil v_{i}/s \right\rceil$ blocks ($\left\lceil \cdot \right\rceil$ is the ceiling function), and the whole genotype data is segmented into ${\textstyle \sum _{i=1}^{n}\left\lceil v_{i}/s \right\rceil }$ blocks. Note that the last block of each chromosome usually contains less than *s* variants. The blocks can be processed in parallel to enhance the computational efficiency.

**Figure 6:**
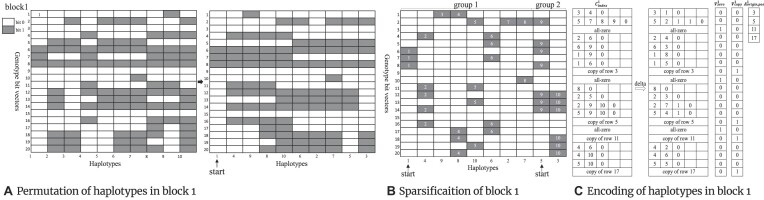
The processing of a genotype data block. (A) Clustering the bit vector blocks: employing a nearest-neighbor algorithm based on Hamming distance for sorting. (B) Sparsification: perform XOR operations on each column of bit data. (C) Sparse encoding: all-zero and copy bit vectors within the block are removed and marked, followed by documenting the specific positions of “1” in the remaining bit vectors of the block.

Each block of genotype data sequentially goes through haplotype clustering, sparsification, and sparse encoding to reach a compact representation. The details of the procedure are provided as follows:

- Haplotype clustering: as shown in Fig. 6A, the haplotypes (columns) within a block are clustered following [15] such that similar columns in terms of Hamming distance are grouped together. The new permutation order of the haplotypes is recorded in an array *P*.
- Sparsification: after the haplotype clustering, every consecutive 8 columns in a block are considered a group for sparsification, since a byte is the minimum unit of data storage, as shown in Fig. 6B. The total Hamming distance between all adjacent columns in a block can be calculated via ${D=\textstyle \sum _{i=1}^{h}d_{i}}$, where $d_{i}$ represents the Hamming distance between columns *i* and $i-1$ if $i \bmod 8 \ne 1$; otherwise, $d_{i}$ is the Hamming weight of column *i* (the number of 1s in column *i*). The sparsity of a block can be evaluated with the number of 1s ${\mho }$ in the block. If ${\mho > D}$, the block is sparsified as follows. Within each column group, if the Hamming distance between a column $X_{i}$ and its predecessor $X_{i-1}$ ($i=2,3,...,8$) is less than the Hamming weight of $X_{i}$, $X_{i}$ is replaced by $X_{i}^{^{\prime }} = X_{i} \oplus X_{i-1}$, where $\oplus$ is an XOR operator. Note that the first column (i.e., $X_{1}$) in each group remains unchanged. Through the above transformation, the sparsity of a group can be reduced as the Hamming weight of $X_{i}^{^{\prime }}$ is not greater than that of $X_{i}$.
- Sparse encoding: after sparsification, there might be a high prevalence of all-zero or duplicate bit vectors. The indexes of the all-zero and duplicate bit vectors in a block are recorded in binary vectors $V_{\rm zero}^{i}$ and $V_{\rm copy}^{i}$, respectively, with the corresponding bits set to “1,” as shown in Fig. 6C. For $V_{\rm copy}^{i}$, the corresponding indexes of the original copies are stored in another integer vector $A_{\rm origin\_pos}^{i}$. Once the positions are properly recorded, the all-zero and duplicate bit vectors are removed from the block. The remaining block becomes sparse and the indexes of bits “1” in each row are stored in an integer vector $C_{\rm index}^{i}$ where “0” is defined as the delimiter of rows, as shown in Fig. 6C. The vector $C_{\rm index}^{i}$ is further encoded into $C_{\rm index\_byte}^{i}$ with delta coding and variable-length codes.

**Figure 7:**
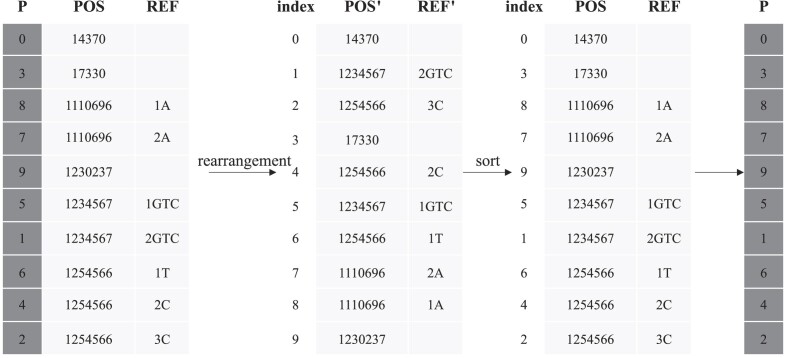
Rearrangement method used to map the array *P* to the POS and REF fields. During compression, the POS and REF fields are rearranged according to *P*. In the recovery phase, the indices are sorted according to the POS values to retrieve *P*.

Due to the haplotype clustering, the haplotypes are repositioned and the original order must be recorded in the array *P* to ensure a lossless reconstruction of the data during the decompression. Nevertheless, if a genotype block contains *h* variants and the POS values are arranged in order, as shown in Fig. 7, *P* can be omitted subject to a corresponding rearrangement of POS and REF values. As illustrated in Fig. 7, given *P*, we can permutate the POS and REF values accordingly such that the information of *P* is encoded in the rearranged POS and REF values. To recover *P*, we can simply sort the rearranged POS values back to the original order and record the permutation. Note that we could also apply delta encoding to the POS values and store the array *P* as it is, yet the space reduction in delta encoding of POS values cannot counteract the extra space required to store *P*. In the last genotype block, where the number of variants is usually not equal to *h, P* is plainly stored with variable byte encoding and the corresponding POS values are stored with delta encoding.

To improve the compression ratio while also maintain query speed, the genotype blocks are further merged into chunks. We adopt a chunk size of $l=65536$ variants, that is, each chunk consists of $m = \left\lfloor l/s \right\rfloor$ blocks ($\left\lfloor \cdot \right\rfloor$ is the floor function). The data of a single chromosome *i* are divided into $\left \lceil \left\lceil v_{i}/s \right \rceil/m \right \rceil$ chunks, and the entire genotype data are finally packed into ${\textstyle \sum_{i=1}^{n}} \left\lceil \left\lceil v_{i}/s \right\rceil/m \right \rceil$ chunks. The chunks are compressed with the general-purpose compressor BSC.

### Compression of other data fields

The INFO and FORMAT fields encompass a variety of subfields of phasing information for genotypes. Each subfield, along with the phasing data (except for the genotype itself), is divided into blocks of 8 MB and compressed using the BSC algorithm.

**Figure 8:**
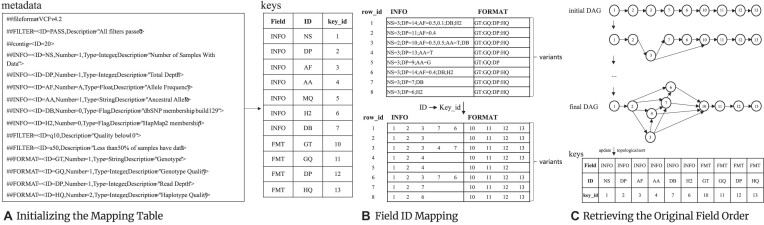
Obtaining the true order of INFO and FORMAT subfield IDs. (A) Constructing a “keys” lookup table. (B) Mapping the IDs of each variant’s INFO and FORMAT fields to their corresponding key_ids. For example, for the variant line “row_id=1,” where the IDs in the INFO field is “NS,” “DP,” “AF,” “DB,” and “H2,” the mapped sequence of “key_id” is “1,2,3,7,6.” (C) Constructing a DAG based on the order of “key_id.”

In a VCF/BCF file, determining the actual order of subfields is challenging when their order specified in the metadata section does not match their actual occurrence in the variant rows. To address this discrepancy, as shown in Fig. 8A, we employ the HTSlib library [29] to parse the metadata and systematically extract the IDs for the INFO and FORMAT subfields. The IDs are then methodically cataloged in a “keys” table, which includes the “Field,” “ID,” and their corresponding “key_id” obtained by HTSlib. Based on the “keys” table, the IDs of the INFO and FORMAT subfields in each variant can be mapped to a string of “key_id,” as shown in Fig. 8B. We then introduce a directed acyclic graph (DAG) to record the “key_id” strings. As shown in Fig. 8C, an initial DAG is constructed with the first “key_id” string recorded in Fig. 8B. Afterward, the DAG is incrementally expanded with next “key_id” string. This process is repeated until all “key_id” strings are incorporated into the DAG. The final DAG is stored with a map data structure and the original “keys” table can be retrieved with a topological sort of the final DAG. As such, lossless decompression of the INFO and FORMAT fields in the variant data is guaranteed. The field values are organized into separate data streams according to their field type. Each data stream is then divided into blocks of size 8 MB to undergo BSC compression.

### Decompression and query

Downstream applications of VCF files are primarily focused on genotype analysis with analytical tools like VCFtools, BCFtools, and PLINK. Most existing VCF compression tools were designed to support only VCF and BCF formats. GSC implements lossless compression and 2 modes of decompression (i.e., lossless and lossy modes). In lossless mode, GSC recovers the original file, whereas in the lossy mode, it retains only the fixed data fields and the genotype data. Both modes enable the decompression of VCF/BCF formats, whereas the lossy mode also supports PLINK binary format.

**Figure 9:**
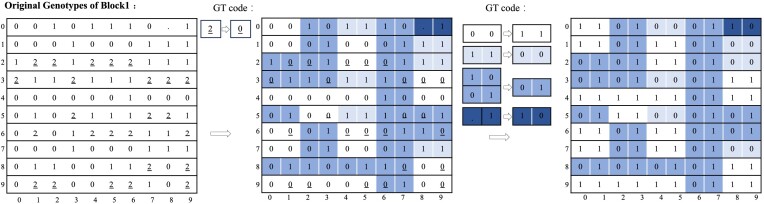
Conversion of genotype data of GSC to “bed” format.

The PLINK binary format (i.e., “bed” format) does not include multiallelic genotypes. Particularly, genotypes are represented as homozygous (0|0 and 1|1), heterozygous (1/0), and missing genotypes (0/1), where “0” denotes a minor allele and “1” a major allele. However, in VCF/BCF files, after preprocessing for multiallelic genotypes, genotypes are denoted as homozygous (0|0 and 1|1), heterozygous (1/0 and 0/1), and missing genotypes (including “.”), with “0” indicating a major allele and “1” a minor allele. To convert the data into “bed” format, we record the second type of nonreference allele “2” back to “0” and construct a mapping table based on the genotype variations (as shown in Fig. 9).

Random access of variants and/or samples is supported by GSC in decompression with specified conditions, including decompression mode, chromosome ID, position range within the chromosome, sample(s), ID of the variant, range of quality values, the minimum/maximum count/frequency of alternate allele among selected samples, and the maximum number of variant sites to decompress. GSC offers options for both variant-based and sample-based queries.

In variant-based query, given the queried variant(s), the corresponding chunks, blocks, and records are identified and decompressed. The chunks, blocks, and records are indexed with a B-tree–like data structure in GSC, such that they could be quickly located. A variant is represented with a 2-bit vector in GSC, which could be a regular vector, empty vector (all zeros), or a duplicated vector. To decompress a regular vector, a decoding procedure is conducted as a reverse of the encoding procedure described in the “Compression of genotype data” section. An empty vector is directly decompressed as all zeros. For duplicated vector, the original copy is identified and recovered according to $V_{\rm copy}^{i}$ and $A_{\rm origin\_pos}^{i}$. Finally, the permutation order recorded in *P* and a byte-level lookup table are used to precisely locate the genotype and position of each variant within the query range.

In sample-based query, if the range of variants is specified, a similar procedure to variant-based query is performed to located the corresponding chunks, blocks, and records. The difference is that only the haplotypes of the queried samples are decompressed. We first determine the position(s) of the byte(s) in the bit vector(s) that encode the haplotypes based on the queried sample names and *P* and then decompress the corresponding bytes to obtain the queried data.

## Availability of Source Code and Requirements

Project name: GSC

Project homepage: https://github.com/luo-xiaolong/GSC

Operating system(s): Linux

Programming language: C++

Other requirements: C++ compiler (e.g., g++)

License: GNU GPL

biotoolsID: gsc_genotype_sparse_compression

RRID: SCR_025071

An archival copy of the code is available via the Software Heritage Archive [30] and the workflow has been registered in workflowhub.eu with a DOI provided in [31].

## Supplementary Material

giae046_GIGA-D-24-00066_Original_Submission

giae046_GIGA-D-24-00066_Revision_1

giae046_GIGA-D-24-00066_Revision_2

giae046_GIGA-D-24-00066_Revision_3

giae046_Response_to_Reviewer_Comments_Original_Submission

giae046_Response_to_Reviewer_Comments_Revision_1

giae046_Response_to_Reviewer_Comments_Revision_2

giae046_Reviewer_1_Report_Original_Submission Jan Voges -- 3/20/2024 Reviewed

giae046_Reviewer_1_Report_Revision_1 Jan Voges -- 5/23/2024 Reviewed

giae046_Reviewer_2_Report_Original_Submission Kirill Kryukov, Ph.D. -- 3/23/2024 Reviewed

giae046_Reviewer_2_Report_Revision_1 Kirill Kryukov, Ph.D. -- 5/19/2024 Reviewed

giae046_Supplemental_File

## Data Availability

The datasets used in this study are publicly available from the following repositories: - The *Mouse Genomes Project* datasets, including SNP and indel information across various mouse strains, were downloaded from the database  [32]. - The *1000 Genome Project–Phase 1* datasets, featuring integrated call sets of the first phase, were obtained from the database  [33]. - The *1000 Genome Project–Phase 3* datasets, encompassing the comprehensive release of phase 3 data, were accessed via the database  [34]. - The *kgenome* datasets, a consolidated file merging VCF data from chromosomes chr1 through chrX, totaling 23 chromosomes, were downloaded from the database  [35]. Note: The “kgenome.vcf.gz” file represents an integration effort to combine VCF files for easier access and analysis. Please refer to the respective repositories and documentation for detailed information on data usage permissions and restrictions.

## Additional Files

**Supplementary Data Section 1**. Information of the Compression Tools.

**Supplementary Data Section 2**. Datasets.

**Supplementary Data Section 3**. Experimental Environment.

**Supplementary Data Section 4**. Additional Results.

## Abbreviations

CMUE: compression memory usage effectiveness; CR: compression ratio; CS: compression speed; DMUE: decompression memory usage effectiveness; DS: decompression speed; DAG: directed acyclic graph; XOR: exclusive Or; KB: kilobyte; GT: genotype; GSC: genotype sparse compression; PBWT: positional Burrows–Wheeler transform; WGS: whole-genome sequencing.
