# Supplementary material for: GSC: efficient lossless compression of VCF files with fast query
Source: Gigascience. 2024 Jul 19;13:giae046. doi: 10.1093/gigascience/giae046 (PMC11258903; doi:10.1093/gigascience/giae046)
Supplement: giae046_GIGA-D-24-00066_Revision_1 [file giae046_giga-d-24-00066_revision_1.pdf]

GigaScience

GSC: Efficient lossless compression of VCF files with fast query

--Manuscript Draft--

**Manuscript Number:**	GIGA-D-24-00066R1
**Full Title:**	GSC: Efficient lossless compression of VCF files with fast query
**Article Type:**	Technical Note
**Funding Information:**	National Key Research and Development Program of China (2022YFF1202104) — Prof. Zexuan Zhu<br>National Natural Science Foundation of China (61871272) — Prof. Zexuan Zhu<br>Guangdong Bigdata Engineering Technology Research Center for Life Sciences (N.A.) — Dr. Yong Zhang
**Abstract:**	Background: With the rise of large-scale genome sequencing projects, genotyping of thousands of samples has produced immense Variant Call Format (VCF) files. It is becoming increasingly challenging to store, transfer and analyze these voluminous files. Compression methods have been used to tackle these issues, aiming for both high compression ratio and fast random access. However, existing methods have not yet achieved a satisfactory compromise between these two objectives.<br>Findings: To address the aforementioned issue, we introduce GSC (Genotype Sparse Compression), a specialized and refined lossless compression tool for VCF files. In benchmark tests conducted across various open-source datasets, GSC showcased exceptional performance in genotype data compression. Compared with the industry's most advanced tools namely GBC and GTC. GSC achieved compression ratios that were higher by 26.9%–82.4% over GBC and GTC on the datasets, respectively. In lossless compression scenarios, GSC also demonstrated robust performance, with compression ratios 1.5–6.5x greater than general-purpose tools like gzip, zstd, and BCFtools—a mode not supported by either GBC or GTC. Achieving such high compression ratios did require some reasonable trade-offs, including longer decompression times, with GSC being 1.2–2x slower than GBC, yet 1.1–1.4x faster than GTC. Moreover, GSC maintained decompression query speeds that were equivalent to its competitors. In terms of RAM usage, GSC outperformed both counterparts. Overall, GSC's comprehensive performance surpasses that of the most advanced technologies.<br>Conclusion: GSC balances high compression ratios with rapid data access, enhancing genomic data management. It supports seamless PLINK binary format conversion, simplifying downstream analysis.
**Corresponding Author:**	Zexuan Zhu<br>Shenzhen University<br>Shenzhen, CHINA
**Corresponding Author Secondary Information:**	
**Corresponding Author's Institution:**	Shenzhen University
**Corresponding Author's Secondary Institution:**	
**First Author:**	Xiaolong Luo
**First Author Secondary Information:**	
**Order of Authors:**	Xiaolong Luo<br>Yuxin Chen<br>Ling Liu<br>Lulu Ding

Powered by Editorial Manager® and ProduXion Manager® from Aries Systems Corporation

	Yuxiang Li
	Shengkang Li
	Yong Zhang
	Zexuan Zhu
**Order of Authors Secondary Information:**	
**Response to Reviewers:**	Dear Editor and Reviewers,<br><br>Thank you for the valuable comments which greatly improve the quality of our manuscript. According to the comments, we have correspondingly revised our manuscript and confirmed that all the comments are well handled. It is noted that the corresponding revisions in the manuscript are highlighted in blue font. The point-by-point response is provided as follows.<br><br>Editor:<br>Comment 1: Major comments of the reviewers include the need for more thorough and wider benchmarking and comparisons to state-of-the-art methods (reviewer #1) and the need to fix a number of problems when compiling and running the software (reviewer #2), among other concerns. In addition, please register any new software application in the bio.tools and SciCrunch.org databases to receive RRID (Research Resource Identification Initiative ID) and biotoolsID identifiers, and include these in your manuscript. Computational workflows should be registered in workflowhub.eu and the DOIs cited in the relevant places in the manuscript. These will facilitate tracking, reproducibility and re-use of your tool.<br><br>Response:<br>Many thanks for handling our manuscript. We have revised the manuscript and improved the experimental study according to the suggestions of the reviewers. The compiling and running issues of the software also have been fixed. We have registered our new software application in the bio.tools and SciCrunch.org databases. The RRID (SCR_025071) and biotoolsID (gsc_genotype_sparse_compression) identifiers of our tool have been provided in our manuscript. We also have made attempt to register the workflows in workflowhub.eu. Unfortunately, our application got stuck with unknown reason. We have contacted the administrator but currently there is no reply. We will cite the DOIs once we get through the registration.<br><br>Reviewer #1:<br>Minor comments:<br>Minor comment 1: - Table 4: The "original size" columns should not be sub-columns of the compression ratio columns (makes no semantic sense).<br><br>Response:<br>Thank you for pointing this out. We have revised the table to make "original size" a separate column.<br><br>Minor comment 2: Figure 3: It is unclear how the GT fields relate to the fixed fields. The information that fixed and other fields are divided into blocks is missing. Are the remaining fixed fields also sorted by P? Also, please clarify in the text how the permutation indices are mapped to the variants when the number of rows and columns in a block is different. Also, the POS field is originally sorted in ascending order, which can be efficiently encoded by first transforming it using delta encoding. Please clarify why the POS needs to be reordered first in this way and whether there is a performance penalty compared to storing the P as it is.<br><br>Response:<br>Many thanks for the suggestion. In the proposed GSC, only the POS and REF fields are reordered according to the permutation order of GT that is recorded in the array P, while the remaining fixed fields are unchanged. We have modified Figure 4 (previous Figure 3) to make this clear.<br><br>Regarding the storage of POS and P, there could be two solutions. In the first one, we could apply delta encoding to the POS field and store the array P as it is. Delta

encoding is indeed an effective method to reduce the space of storing the POS field since it is sorted in ascending order. However, it requires extra space to store P. In the second solution, the POS field could be reordered according to P and only the reordered POS is stored, as P could be recovered from the reordered POS as shown in Figure 7. We have compared the two solutions in Section ‘Effects of the key components’, Page 3, and found the second one can achieve better compression ratio. The reason is that recording the reordered POS in the second solution is equivalent to storing the array P in the first solution, yet the second one is exempt from storing the delta encoded POS.

In a block where the number of rows and columns is different (only occurring as the last block), the permutation indices in P are stored directly with variable-length byte encoding and the corresponding POS field is stored with delta encoding. We have added detailed description in the right column of Page 6.

Minor comment 3: - Figure 5a: The white right arrow is misplaced and barely visible.

Response:
Thank you for pointing this out. We have repositioned the arrow and enhanced its contrast for better visibility.

Minor comment 4: - Figure 5c: "encoding" -> "Encoding"

Response:
Thank you for pointing this out. We have fixed it in this revision.

Minor comment 5: - Figure 6: It should be made clear in the figure (caption) that the rearrangement sorts the rows by P and that the sort sorts the rows by POS.

Response:
Thank you for your suggestion. We have accordingly revised the figure (now Figure 7) caption to better explain the role of the arrays and the sequence of operations during the compression and recovery phases.

Minor comment 6: - Figure 7b: "FROMAT" -> "FORMAT"

Response:
Thank you for pointing this out. We have fixed the typo.

Minor comment 7: - Figure 7b: The order/arrangement of the tables is unclear.

Response:
Thank you for your suggestion. We have revised the figure caption to better describe how each table relates and interacts within the figure.

Minor comment 8: - Figure 7b: Please explain where and how the values of each field are stored.

Response:
Many thanks for the suggestion. Figure 8(b) (previous Figure 7b) is introduced to illustrate how IDs in INFO and FORMAT subfields transfer to key_id strings. The order of the IDs is stored in the DAG as shown in Figure 8(c). The field values are organized into separate data streams based on their field type. Each data stream is then divided into blocks, with each block being 8MB in size, to facilitate efficient storage. We have added description in the left column of Page 7.

Major comments:

Comment 1: - Please address the limitations of the proposed approach.

Response:
Thank you for your valuable suggestion. Despite the promising performance of GSC, there are some limitations of GSC. It cannot handle datasets containing no genotype information, such as the ExAC and gnomAD datasets. This limitation is inherent to the

Powered by Editorial Manager® and ProduXion Manager® from Aries Systems Corporation

design of GSC, which features a high degree of coupling between genotype information and the POS fields, and is specifically optimized for the compression and retrieval of genotype data. It deserves future efforts to explore new methodologies and algorithms capable of incorporating and efficiently compressing these critical datasets. We have added discussion on the limitations and future work in the ‘Discussion’ section.

Comment 2: Please provide an evaluation of compression/decompression times and memory consumption, including information about multi-threading. This information is critical to understanding the scalability of the compared methods.

Response:
Thank you for the suggestion. We have provided the equivalent evaluation in our manuscript in Figure 1(b), where the metrics CS (Compression Speed) and DS (Decompression Speed) along with CMUE (Compression Memory Usage Efficiency) and DMUE (Decompression Memory Usage Efficiency) are represented in a radar chart. These indicators are designed to offer a comprehensive view of the performance and efficiency of each method under study. We also provide more detailed information of the test results in Section 4 of the Supplementary data. The experiments were conducted on a single processing thread. The configurations of the compared methods are provided in Section 3 of the Supplementary data. We have revised the description in the ‘Compression performance’ section to make this clear.

Comment 3: To better evaluate GSC's performance, you should evaluate GSC on larger and more recent datasets such as HRC, ExAC, and gnomAD, which may contain other fields.

Response:
Thank you very much for the valuable suggestion. The HRC dataset is managed under the stewardship of the Sanger Institute, which imposes strict access controls. Despite our efforts to obtain permission, we have not been granted the access to this dataset. The lack of access has unfortunately limited our evaluations on the HRC dataset.

GSC is oriented for conventional VCF files and it is specifically optimized for datasets containing genotype information, which is tightly integrated with POS fields to achieve high compression ratios and efficient data retrieval. Unfortunately, this design makes GSC unsuitable for compressing datasets without genotype data like ExAC and gnomAD. We have discussed this limitation in the ‘Discussion’ section. GSC could probably be modified to handle ExAC and gnomAD by sacrificing some compression performance, yet more efficient methods are more desirable in our future work.

Comment 4: Also, you should compare GSC with state-of-the-art methods such as VCFShark, GenoZip, and XSI for whole VCF file compression.

Response:
Many thanks for the suggestion. We have involved XSI in the comparison with GSC in the compression of genotype data (results shown in Table 1 and Figure 1), and VCFShark and GenoZip in the compression of whole VCF files (results shown in Table 2). The query performance of XSI and GenoZip is also reported in Figure 3.

GSC shown better overall compression and query performance than XSI. Since GSC has to maintain some extra space to support the fast query functionality, it is compression ratio on whole VCF file is slightly inferior to VCFShark and GenoZip. It might be inappropriate to directly compare GSC with fast random accessibility to VCFshark and Genozip, which are not intended to support fast random access. Yet we have provided discussion on the results in the left column of Page 3.

Comment 5:- Section "Introduction": You mention here that the genotype data may only constitute a small part of a VCF file, but later in the paper you elaborate that larger datasets (1000GPip3 and Kgenome) consist primarily of genotype data. Please clarify this.

Response:
Thank you for pointing this out. In the introduction, we aimed to highlight the diverse

Powered by Editorial Manager® and ProduXion Manager® from Aries Systems Corporation

nature of VCF files, which can contain a wide range of data types beyond genotype information, such as annotations, metadata, and non-genotypic variant data. The intent was to emphasize that, while genotype data is a significant component, VCF files are not limited to this type of data alone and can encompass a broad spectrum of genomic information. To avoid causing potential confusion to the reader, we have revised the description in the 'Introduction' Section as follows:

"Most of the above methods primarily focus on the compression of genotype data while disregarding the other annotation data present in VCF files. Given that genotype data might constitute only a portion of VCF files, such as in the first phase of the 1000 Genomes Project dataset, relying solely on genotype compression is insufficient to alleviate the storage and transmission pressures of large VCF files."

Comment 6:- Section "Data Description": It is unclear to me what you mean by to "test the compatibility of GSC". What is the added value of the Kgenome dataset? Why should a method fail on concatenated VCF data, while it works on the individual VCF data?

Response:
Many thanks for the suggestions and apologies for not being able to make it clear in our manuscript.

Our primary goal in testing the compatibility of the GSC tool was to specifically evaluate whether the compressor can handle VCF files containing multiple chromosomes, which are increasingly prevalent in genomics research. As datasets grow in size and complexity, it is essential to ensure that GSC remains compatible with these more complex datasets.

The Kgenome dataset, unlike individual VCF files from the 1000 Genomes Project phase 3 (1000GPip3) that typically contain genomic data for a single chromosome, comprises merged VCF files for all 22 autosomes and the X chromosome. This integration significantly elevates the dataset's complexity and size, making it an ideal benchmark for testing the compression capabilities of GSC on concatenated datasets. It also allows us to evaluate GSC's proficiency in handling complex, real-world genomic datasets.

Some compression tools, such as PBWT, are designed with the assumption that data follows the structure of single chromosomes. When faced with concatenated VCF files that include data from multiple chromosomes, these tools may struggle to manage the increased complexity and volume of data. This limitation underscores the importance of developing compression tools that can adapt to the characteristics of concatenated datasets.
We have provided revised description in the Section "Data Description" (Page 2) to clarify this point.

Comment 7:- Section "Findings"/Table 1: In my opinion, the compression ratio comparison is biased because the tools use different block sizes, which usually has a non-negligible impact on the compression ratios obtained. This may also be the reason why GSC largely outperforms GTC and other methods across all datasets. Please investigate and clarify this, e.g., by presenting results obtained using similar block sizes for all methods. In addition, it would be interesting and important to see at what block size GSC's performance saturates.

Response:
Thank you for the valuable suggestion. To avoid the potential biases introduced by using different block sizes, we have re-run the experiments with GSC, GBC, and GTC configured the same block size. The new results are updated to Table 1 and Figure 1. The other methods including BCFtools, XSI, and PBWT do not support custom block size. Their configurations were not changed.

Currently, the block size of GSC is determined based on the number of samples, which maximized the redundancy reduction in the haplotype clustering and sparsification. Nevertheless, exploring the performance of GSC with different block sizes could provide further insights into the scalability of GSC and more flexibility to the user. We

Powered by Editorial Manager® and ProduXion Manager® from Aries Systems Corporation

have added discussions on this point in the ‘Discussion’ Section.

Reviewer #2:
Comment 1: p2: "All compressors were run with a minimum two threads" - It's not clear if all compressors were using the same number of threads, and why you mention the minimum instead of a maximum number of threads. I also think it would be informative to see single-threaded performance comparison.

Response:
Many thanks for the suggestion. We have re-run the experiments and all compressors were configured to use a single thread. We have provided the following description in the Section "Findings/Compression performance" of the manuscript (Page 2):
“All compressors were run with a single thread on the same operating system. The detailed software and hardware configurations are provided in Section 3 of the Supplementary data.”

Comment 2: p4: "Afterward, the processed blocks are merged and encoded with general-purpose compressor BSC" - It is interesting what considerations determined the choice to use BSC, and if other compressors / libraries were considered. E.g., brotli, zstd, lz4, lzma might be valid choices for some of the tasks in this context. It may be good to explain the reasons for choosing BSC in the text.

Response:
Thank you very much for the suggestion. The selection of backend encoder is critical to the performance of GSC. To investigate the effects of using different compressors as suggested, we have evaluated GSC with different compressors including brotli, zstd, lz4, lzma, and BSC. The comparison results in terms of Compression Ratio, Compression and Decompression Speed, and Memory Use Efficiency in 1000GPip1, 1000GPip3, and Mgp datasets have been provided in Figure 2. brotli achieved the highest compression ratio but was slower in terms of compression speed. lz4 excelled in compression speed and memory efficiency at a cost of lower compression ratio. lzma, zstd, and BSC obtained the better overall performance than brotli and lz4. Among them, BSC achieved the best compromise in terms of all performance metrics, which justifies our selection of BCS as the backend compressor of GSC. We have provided the results and discussions of this comparison study in the ‘Effects of the key components’ Section.

Comment 3: I recommend adding the zstd compressor to the comparison, as a more relevant example of a modern general purpose compressor.

Response:
Thank you for the recommendation. We have included zstd in the comparison study and the results are presented in Tables 1 and 2 as well as Figure 1.

Comment 4: It's not clear if GSC supports a streaming mode of operation, i.e., reading from standard input and writing to standard output. If it's supported, it's better to mention it in both the text and the github page, with example command lines. If it's not supported, I recommend adding such support, as it will greatly help integrating GSC into pipelines.

Response:
Thank you very much for pointing this out. GSC indeed supports streaming mode from standard input to standard output. We have updated accordingly in both the text (the ‘Discussion’ Section) and the GitHub page, which now includes an example ‘./gsc compress -i sample-8.vcf > sample-8.vcf.gsc’ to guide the users on utilizing GSC within a streaming context.

Comment 5: Compiling GSC produces many warnings, which does not inspire confidence in code quality. Please consider fixing all warnings. (I used "g++ (GCC) 12.3.1 20230508 (Red Hat 12.3.1-1)" on Fedora 37).

Response:
Many thanks for pointing this out. We have resolved the issues by meticulously revising

Powered by Editorial Manager® and ProduXion Manager® from Aries Systems Corporation

	our code to ensure it adheres to the best practices and standards in C++ programming. These revisions were tested using the same version of GCC you mentioned (GCC 12.3.1 on Fedora 37) to ensure that no warnings are produced during compilation. The latest version of GSC has been updated on our GitHub page.<br><br>Comment 6: Command line example from github page: "gsc compress <options> [out_file] [in_file]" - It's not a good idea to allow specifying the input and output files this way, as it looks there might be a risk of swapping their position around, accidentally overwriting the input file. Instead I recommend requiring "--in [in_file]" and "--out [out_file]", to make it clear which file is the input and which one is the output. It will also make it easier to read GSC command lines in pipelines.<br><br>Response:<br>Thank you very much for the constructive feedback regarding the syntax of the command line interface. Following your recommendation, we have updated the command line interface to require explicit flags for input and output files. The new syntax now uses "--in [in_file]" and "--out [out_file]" to clearly designate the input and output files, respectively. We have updated the documentation on our GitHub page to reflect these changes and provide clear guidance for our users.<br><br>Comment 7: After building GSC, at first I could not make it work for compression. "./gsc" shows the help message mentioning both "compress" and "decompress" modes. However, trying compression on some of my data with "./gsc compress sample-8.vcf.gsc sample-8.vcf" did nothing and somehow just shown a help message for the decompression command. Adding "--out" helped, i.e., this command worked: "./gsc compress --out sample-8.vcf.gsc sample-8.vcf". I suggest improving the github documentation to make it clear how to run the command.<br><br>Response:<br>Thank you for pointing this out. We have conducted a thorough review and testing of the command line interface, specifically focusing on the issues you encountered with the "compress" command. We have identified and corrected the issue that caused the incorrect display of help messages and potentially hindered the execution of compression commands.<br>We have also tested the compression functionality using the example file "sample-8.vcf" as you mentioned, and we can confirm that the issue has been resolved. To prevent any future confusion, we have updated the GitHub documentation to clearly illustrate the correct usage of the compression command, especially emphasizing the necessity of the "--out" flag for clarity. The updated documentation now includes more explicit instructions and examples to ensure that users can easily understand and correctly use GSC without encountering similar issues.<br>Comment 8: After compression completed, unexpectedly there was no output file "sample-8.vcf.gsc" produced. Instead there were two files named "sample-8.vcf.gsc.dbs" and "sample-8.vcf.gsc.gti". I have to say that this is an unfortunate choice. Multi-files archives complicate file and data management, and make it harder to integrate this compressor into pipelines. E.g., imagine a scenario of taking a vcf file, compressing it with gsc, and immediately streaming to a remote server. Or vice versa, streaming gsc-compressed data from remote machine, decompressing it on the fly, and immediately streaming the data to the next analysis step. These usages are not possible, or at least inconvenient, when a gsc archive consists of multiple files. I hope you can reconsider and switch to a single-file archive format.<br><br>Response:<br>Thank you very much for the suggestion. We have revised the code to generate a single-file archive, using only the ‘.gsc’ extension. The updated version with these modifications has been thoroughly tested to ensure that it maintains the same level of compression efficiency and integrity. We have also updated the GitHub documentation to reflect these changes and provide clear guidance on using the new single-file format.<br><br>Comment 9:. Decompressing my data back to vcf with the command "./gsc decompress --out sample-8.vcf sample-8.vcf.gsc" did not produce a file "sample-8.vcf", which I expected it to produce. Instead it produced a file "sample-8.vcf.vcf". Please avoid modifying user-supplied file names and use them exactly as they are.

	Response:<br>Thank you for pointing out the issue. We apologize for the inconvenience caused by the unexpected modification of the output file name. We have identified an oversight in the decompression command logic that led to the addition of an extra file extension. We have corrected this issue to ensure that the tool uses the file names exactly as specified by the user without any alterations.<br><br>Comment 10: The decompression command mentioned above crashed with a message "Segmentation fault (core dumped)". It produced partial vcf output, but not the entire file. I uploaded my test data so you can use it for testing (zstd-compressed): https://biokirr.com/Shared-Data/GSC/ . Since this is the first dataset I tried, this leaves a bad impression. It should be obvious that a data compressor must be rock-solid and reliable.<br><br>Response:<br>Apologies for the difficulties you encountered while using GSC. We greatly appreciate your effort in testing our software and sharing your feedbacks. We have resolved the issue and conducted extensive testing across various datasets, including the data you kindly uploaded, to ensure that no similar issues remain. We have also updated our GitHub documentation and implementation to reflect these changes and improvements. Your insights have been invaluable to enhancing the robustness of our tool, and we are truly grateful for your contribution.
**Additional Information:**	
**Question**	**Response**
Are you submitting this manuscript to a special series or article collection?	No
**Experimental design and statistics**<br><br>Full details of the experimental design and statistical methods used should be given in the Methods section, as detailed in our Minimum Standards Reporting Checklist. Information essential to interpreting the data presented should be made available in the figure legends.<br><br>Have you included all the information requested in your manuscript?	Yes
**Resources**<br><br>A description of all resources used, including antibodies, cell lines, animals and software tools, with enough information to allow them to be uniquely identified, should be included in the Methods section. Authors are strongly encouraged to cite Research Resource Identifiers (RRIDs) for antibodies, model organisms and tools, where possible.	Yes

Have you included the information requested as detailed in our Minimum Standards Reporting Checklist?	
**Availability of data and materials**<br><br>All datasets and code on which the conclusions of the paper rely must be either included in your submission or deposited in publicly available repositories (where available and ethically appropriate), referencing such data using a unique identifier in the references and in the “Availability of Data and Materials” section of your manuscript.<br><br>Have you have met the above requirement as detailed in our Minimum Standards Reporting Checklist?	Yes

Powered by Editorial Manager® and ProduXion Manager® from Aries Systems Corporation

Manuscript

Click here to access/download;Manuscript;GSC.pdf

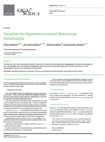

GigaScience, 2024, 1–9

doi: xx.xxxx/xxxx
Manuscript in Preparation
Technical Note

TECHNICAL NOTE

GSC: Efficient lossless compression of VCF files with fast query

Xiaolong Luo[1], Yuxin Chen[2,3,4], Ling Liu[5], Lulu Ding[1], Yuxiang Li[2,3,4], Shengkang Li[2,3,4], Yong Zhang[2,3,4,*] and Zexuan Zhu[6*]

[1]College of Computer Science and Software Engineering, Shenzhen University, Shenzhen 518060, China and [2]BGI Research, Wuhan 430074, China and [3]BGI Research, Shenzhen 518083, China and [4]Guangdong Bigdata Engineering Technology Research Center for Life Sciences, BGI Research, Shenzhen 518083, China and [5]Guangzhou Institute of Technology, Xidian University, Guangzhou 510555, China. and [6]National Engineering Laboratory for Big Data System Computing Technology, Shenzhen University, Shenzhen 518060, China.

[*]To whom correspondence should be addressed. E-mail: zhangyong2@genomics.cn, zhuzx@szu.edu.cn

Abstract

Background: With the rise of large-scale genome sequencing projects, genotyping of thousands of samples has produced immense Variant Call Format (VCF) files. It is becoming increasingly challenging to store, transfer and analyze these voluminous files. Compression methods have been used to tackle these issues, aiming for both high compression ratio and fast random access. However, existing methods have not yet achieved a satisfactory compromise between these two objectives.
Findings: To address the aforementioned issue, we introduce GSC (Genotype Sparse Compression), a specialized and refined lossless compression tool for VCF files. In benchmark tests conducted across various open-source datasets, GSC showcased exceptional performance in genotype data compression. Compared with the industry's most advanced tools namely GBC and GTC. GSC achieved compression ratios that were higher by 26.9%–82.4% over GBC and GTC on the datasets, respectively. In lossless compression scenarios, GSC also demonstrated robust performance, with compression ratios 1.5–6.5x greater than general-purpose tools like gzip, zstd, and BCFtools—a mode not supported by either GBC or GTC. Achieving such high compression ratios did require some reasonable trade-offs, including longer decompression times, with GSC being 1.2–2x slower than GBC, yet 1.1–1.4x faster than GTC. Moreover, GSC maintained decompression query speeds that were equivalent to its competitors. In terms of RAM usage, GSC outperformed both counterparts. Overall, GSC's comprehensive performance surpasses that of the most advanced technologies.
Conclusion: GSC balances high compression ratios with rapid data access, enhancing genomic data management. It supports seamless PLINK binary format conversion, simplifying downstream analysis.

Key words: VCF/BCF files; lossless compression; rapid random access.

Introduction

In recent decades, continuous advancements in technology and cost reductions in sequencing have resulted in a significant increase in large-scale sequencing projects, leading to a rapid growth of genotypic data. Currently, the Variant Call Format (VCF) is the most commonly used format for storing DNA polymorphism data, encompassing single nucleotide polymorphisms, insertions, deletions, structural variations, and extensive annotations [1]. However, as a text-based format, VCF files occupy substantial storage space due to inherent redundancy. With the escalation of large-scale sequencing projects, the number and size of VCF files experience a dramatic surge. For example, the 1000 Genomes Project [2]and the analysis of whole-genome sequencing (WGS) of 150,119 individuals from the UK Biobank [3] generated VCF files in the hundreds of terabytes. Considering future projects that could scale to millions

Compiled on: May 16, 2024.
Draft manuscript prepared by the author.

of samples, storing, transferring and analyzing VCF files present increasingly challenging tasks. To address these issues, general-purpose compression methods, as well as more compact binary formats like BCF [4], have been widely employed. However, the compression ratios provided by these methods or formats are inadequate for handling large WGS genotype data contained in VCF files.

In recent years, numerous specialized compression algorithms for VCF files have emerged to improve the efficiency of storage, maintenance, and transmission. These algorithms can be broadly classified into two categories.

The first category primarily focuses on achieving high compression ratios, without significant consideration for the random-accessibility of the compressed data. For instance, GTShark [5] and SAV [6] employ positional burrows–wheeler transform (PBWT) [7] to reposition the variant record data. This enables the identification of more data redundancy and facilitates more efficient genotype compression. VCFShark [8], an extension of GTShark, enhances the compression of the entire VCF file by incorporating special processing of the variant descriptive information. Genozip [9] also offers a lossless compression solution for VCF files, considering both genotype and additional annotation information. It also supports basic random access to the compressed data. Although these methods achieve superior compression ratios, they may not provide rapid random access to genotype data, which could be vital for subsequent analyses.

The second category involves the partitioning and reorganization of genotype data in order to achieve a balance between high compression ratios and efficient genotype retrieval. For instance, the aforementioned study introducing PBWT [7] also utilizes PBWT and run-length encoding techniques to greatly improve the compression ratio of genotype data. This approach also facilitates efficient matching of haplotypes in terms of time and space. GQT [10] optimizes the retrieval of individual genetic variations by transposing genotype data and applying word-aligned hybrid compressed bitmap indices. BGT [11] enables queries of genotypes and variants and efficiently manages complex variants in VCF files by separating sample phenotypes, site annotations, and genotypes, and utilizing a 2-bit integer matrix combined with PBWT compression technology. GTRAC [12] achieves efficient compression of VCF files by building variant dictionaries and compressing binary matrices, and provides specific query functionalities on the compressed data. SeqArray [13] offers users various efficient compression options and data access capabilities by utilizing the LZMA compression algorithm [14]. GTC [15] improves compression ratios and query speeds by rearranging genotype data in blocks and utilizing run-length and Huffman coding techniques. XSI [16] employs a hierarchical block compression strategy that leverages sparse coding, word-aligned hybrid encoding, and PBWT to achieve efficient genotype data compression. It uses BCF format to store the variant annotation information for random data retrieval. GBC [17] features partitioning and block segmentation, an efficient storage structure, and a parallel algorithm that significantly accelerates the query speed. GVC [18] achieves compression of gene sequence variations with random access capability through the use of binarization, joint row- and column-wise sorting of variation blocks, and the efficient image compression codec JBIG [19]. Most of the above methods primarily focus on the compression of genotype data while disregarding the other annotation data present in VCF files. Given that genotype data might constitute only a portion of VCF files, such as in the first phase of the 1000 Genomes Project dataset [2], relying solely on genotype compression is insufficient to alleviate the storage and transmission pressures of large VCF files.

In this article, we introduce GSC (Genotype Sparse Compression), a specialized and refined lossless compression tool designed for handling entire VCF files. GSC efficiently compresses both genotype data and annotation information within VCF files independently, enabling fast and diversified variant querying. It achieves exceptional compression ratios for both genotype data and the entire VCF file, while maintaining rapid data querying capabilities. Additionally, the compressed files generated by GSC can seamlessly be converted into the binary format required by PLINK, a widely used tool for genome-wide association studies [20]. This integration significantly accelerates downstream analysis. GSC offers a promising solution for storing VCF files by striking a fine balance between compression efficiency, random-access capability, and support for downstream analysis.

Data Description

To evaluate the performance of GSC, we selected datasets from Phase 1 (1000GPip1: 1,092 samples, 39,707,426 variants) and Phase 3 (1000GPip3: 2,054 samples, 84,740,066 variants) of the 1000 Genome Project [21], as well as the dataset from sequencing project Mgp [22]. Each dataset comprises multiple VCF files, with each file containing data of a single chromosome. We merged all VCF files from the 1000GPip3 dataset into a single VCF file named Kgenome, specifically to evaluate whether the compressor can handle VCF files containing multiple chromosomes. More details of the datasets provided in Section 2 of the Supplementary Data.

Findings

Compression performance

To evaluate the compression performance of GSC, we conducted a comparison between GSC and other representative state-of-the-art random-accessible VCF compressors including GBC [17], GTC [15], XSI [16], and PBWT [7]. In addition, the general-purpose compressor gzip [23], zstd [24], and BCFtools [4] were also involved as the baselines. To ensure the fairness of the comparison, especially in the mode focusing solely on genotype data compression, we excluded all subdomains from the INFO and FORMAT data fields, except for the 'GT' subdomain. GBC, GTC, and GSC all compress the data in a block-wise fashion. To avoid the potential biases introduced by using different block sizes, we configured the three methods with the same block size. All compressors were run with a single thread on the same operating system. The detailed software and hardware configurations are provided in Section 3 of the Supplementary data.

The compression ratios (original data size/compressed data size) of the compressors are summarized in Table 1, where the results demonstrate the superiority of GSC to the compared methods. GSC offers a highly competitive compression ratio in genotype data compression. For example, in 1000GPip3 dataset that predominantly comprises genotype data, GSC achieves a compression ratio of 712.07, which is 1.5-5.5x of that of other random-accessible VCF compressors, i.e., GBC, GTC , XSI and PBWT, and 8-10x of that of the general-purpose compressor gzip, zstd and BCFtools. We also explored the efficiency of the compressors across different chromosomes within 1000Gpip3 as shown in Fig. 1 (a). GSC performs consistently across different chromosomes. PBWT and XSI failed to compress the dataset of the ChrX chromosome that contains genotypes of varying ploidy.

We further conducted an evaluation of the overall performance of the compared methods in terms of Compression Ratio (CR), Compression Speed (CS), Decompression Speed (DS), Compression Memory Usage Effectiveness (CMUE), and Decompression Memory Usage Effectiveness (DMUE). The Memory Usage Effectiveness is defined as $1/\log_{10}^{T}$, where T is the peak size of memory (KB) used during compression/decompression. As the radar chart shown in Fig. 1 (b), GSC demonstrates a good compromise performance over all the five metrics, which could be estimated by the area covered on the chart, and superior in terms of compression ratio.

Table 1. Compression Ratios of Genotype Data

Datasets	Variant sites	Original (GB)	gzip	BCFtools	zstd	PBWT	XSI	GTC	GBC	GSC
Mgp	90,310,977	18.57	23.68	25.79	17.10	7.2	14.09	20.41	42.2	**53.06**
1000GPip1	39,707,426	156.49	38.15	57.53	32.81	100.31	68.35	180.37	191.08	**289.80**
1000GPip3	84,740,066	794.36	63.37	88.60	72.53	172.77	435.29	465.92	391.54	**714.11**
Kgenome	84,740,066	785.10	62.32	91.21	71.61	-	-	461.71	392.55	**705.73**

Note: “-” indicates the data cannot be successfully compressed by the corresponding method.

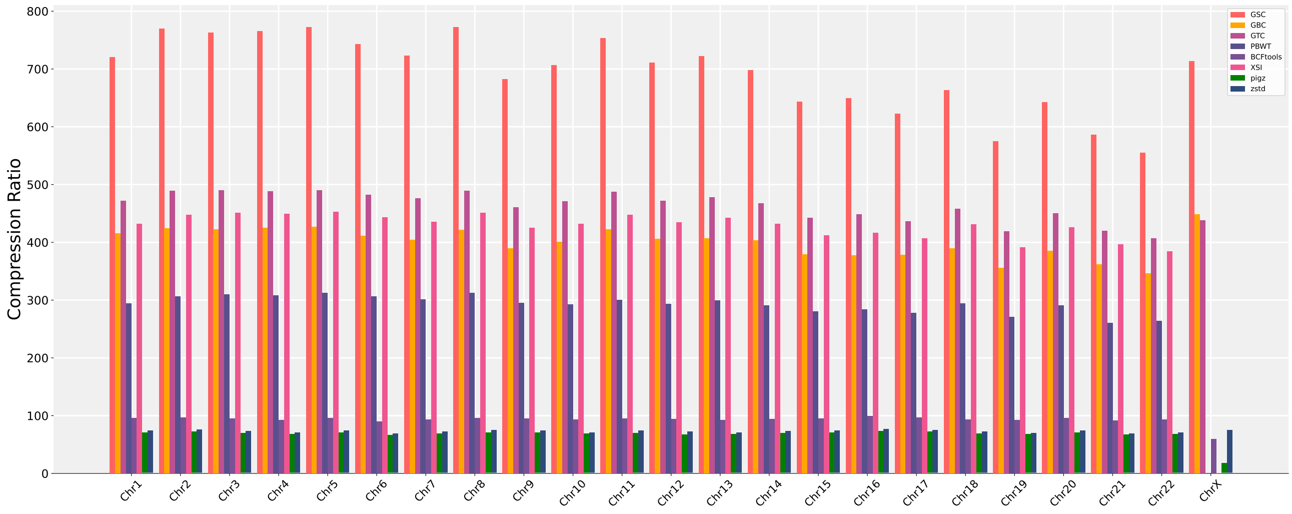

(a) Compression ratios on different chromosomal files

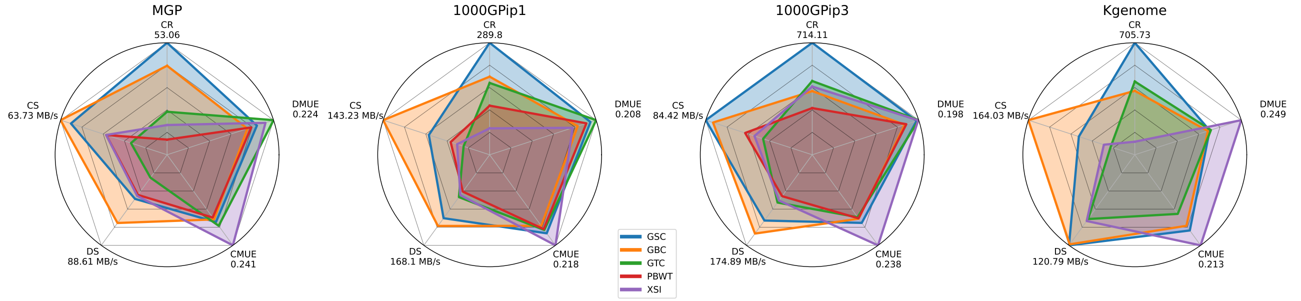

(b) Comprehensive performance of the compression tools. CR: Compression Ratio, CS: Compression Speed, DS: Decompression Speed, CMUE: Compression Memory Usage Effectiveness, and DMUE: Decompression Memory Usage Effectiveness.

Figure 1. Compression results of genotype data. (a) Compression ratios of various tools in the dataset 1000GPip3 across 23 chromosomes files. (b) Comprehensive performance comparison of compressors on datasets Mgp, 1000GPip1, 1000GPip3, and Kgenome.

In addition to the genotype data compression, GSC also supports the compression of a whole VCF file. We compared GSC with the general-purpose compressors including BCFtools, gzip, and zstd, and the specialized whole VCF file compressors, i.e., Genozip and VCFshark, in the compression of the whole VCF files in Table 2. Note that it might be inappropriate to directly compare GSC with fast random accessibility to the other methods that do not well support random access. Yet GSC still achieves significantly better compression ratios than the general-purpose compressors, i.e., BCFtools, gzip, and zstd. Since GSC has to maintain reasonable extra space to support the fast query functionality, its compression ratios are expectably inferior to VCFshark and Genozip, which are not intended to support fast random access. GBC, GTC, and PBWT were not included in this comparison since they cannot handle the whole VCF file.

Effects of the key components

GSC is featured by haplotype clustering and sparsification that leads to repositioning of the haplotypes. As described in Section *Compression of genotype data*, the new permutation order of the haplotypes denoted by an array P is the pivotal information that must be recorded to ensure lossless compression. To record P, in GSC, we reorder the POS field according to P and store merely the reordered POS values (named as reordered mapping scheme). The original POS values and P can be fully recovered from the reordered POS values (as illustrated in Fig. 7). Alternatively, we can apply delta encoding to the POS values and store the array P as it is (named as direct storage scheme). To investigate the effectiveness of the reordered mapping scheme, we compared it with GSC using the direct storage scheme. The experimental results are reported in Table 3, where the reordered mapping scheme shows superior compression performance.

Table 2. Compression Ratios of Whole VCF File

Datasets	Variant sites	Original (GB)	gzip	BCFtools	zstd	Genozip	VCFshark	GSC*
Mgp	90,310,977	182.22	5.28	4.99	5.05	**9.17**	9.16	8.35
1000GPip1	39,707,426	878.37	8.08	6.13	6.05	17.48	**19.82**	17.32
1000GPip3	84,740,066	803.70	67.75	67.12	80.95	547.31	**563.12**	438.31
Kgenome	84,740,066	794.84	67.00	66.38	78.91	549.34	**557.54**	433.40

* GSC enable fast random access, whereas the other methods do not well support random access.

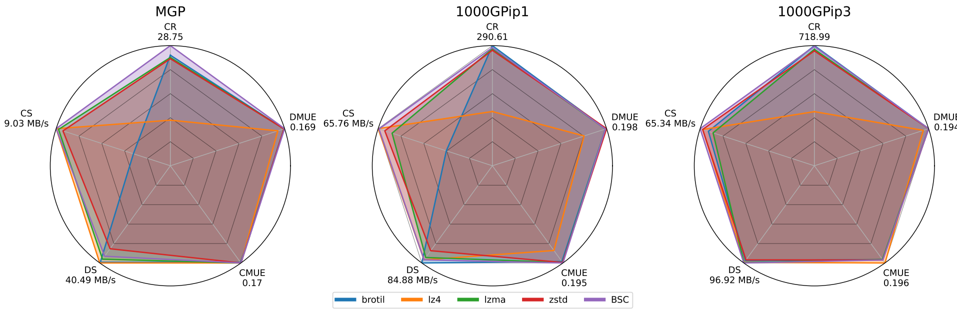

Figure 2. Performance comparison of using different backend compressors in GSC on datasets Mgp, 1000GPip1, and 1000GPip3.

Table 3. Compression Ratios: Direct Storage vs. Reordered Mapping

Datesets	GSC (Direct Storage)	GSC (Reordered Mapping)
MGP	46.89	**53.06**
1000GPip1	277.15	**289.80**
1000GPip3	698.99	**712.07**

As detailed in the Section *Methods*, GSC uses BSC as the backend encoder for the compression of different data streams. The selection of backend encoder is critical to the overall performance of GSC. To investigate the effects of using different compressors as the backend, we evaluated GSC with mainstream compressors including brotli [25], zstd , lz4 [26], lzma [27], and BSC. The comparison results in terms of Compression Ratio, Compression and Decompression Speed, and Memory Use Efficiency in 1000GPip1, 1000GPip3, and Mgp datasets are plotted in Fig. 2. brotli achieved superior compression ratios at the cost of compression speed. lz4 excelled in compression speed and memory efficiency but it obtained lower compression ratios. lzma, zstd, and BSC attained better overall performance than brotli and lz4. Among them, BSC achieved the best compromise in terms of all performance metrics, which justifies our selection of BCS as the backend compressor in GSC.

Query performance

Supporting rapid genotypic extraction is highly demanded in the compression of VCF files. GSC not only provides high compression ratios in both lossless and lossy compression modes, but also enables swift and flexible genotypic querying, a feature not found in most generic compression tools. To evaluate the query performance of GSC, we selected the representative chromosome 1 from dataset 1000GPip3 as the target for genotype querying, which comprises 2,504 samples and 6,468,094 variants. GSC was compared with GBC, GTC, PBWT, BCFtools, XSI, and Genozip in both variant-based and sample-based querying. It is important to note that VCFshark was not included in this comparison as it does not support random access.

Query time for genotypes across various variant ranges using different tools is displayed in Fig. 3(a). The sample size was consistently set to 2,504. In queries of less than 1,000 variant rows, GTC , XSI and BCFtools completed in just a few hundredths of a second., while GBC and GSC took slightly longer time, i.e., up to a tenth of a second. PBWT consumed 1-2 seconds to finish the same query. Genozip tends to be slower than other tools by taking up to 10 seconds. As the query range increased to over 10,000 variant rows, the querying speeds of GSC and GBC exceed that of other methods.

The running time for querying different sample sizes of the methods within a set range of 1,000,000 variant rows are delivered in Fig. 3(b). For queries involving less than 50 samples, GTC and GBC demonstrated comparable efficiency, with the query times ranging from 2 to 7 seconds. GSC, though marginally slower, completed queries for these smaller sample sets in approximately 8 seconds, while PBWT and BCFtools required substantially longer running time, often tens of seconds for equivalent tasks. As the sample count increases, GSC and GBC again show advantage against other tools. Genozip needs to decompress the entire file for queries, showing a consistent query time of around 190 seconds, regardless of the sample size. XSI performed comparably to GSC for single-sample queries but failed when querying multiple samples.

In summary, with larger query ranges, GBC consistently demonstrated the shortest query time, and GSC is the runner-up in both variant-based and sample-based querying. Genozip and BCFtools tend to be less efficient than other tools. For more details of the querying results, the reader is referred to Section 4 in the Supplementary data.

Efficiency of format conversion

To demonstrate the efficiency of format conversion from the output of GSC to PLINK binary format, i.e., 'bed' format, we compared the conversion runtime and memory usage of GSC output to 'bed' vs. VCF to 'bed' using PLINK on Mgp, 1000GPip1, and 1000GPip3 datasets as shown in Fig. 3(c). Note that PLINK does not support direct conversion of VCF containing multi-allelic genotypes to 'bed'. Hence, BCFtools has to be used to preprocess the VCF files for PLINK. In contrast, GSC can efficiently handle multi-allelic genotypes and variant description information during the VCF compression process, i.e., it enables direct conversion of the compressed file to 'bed' format. As depicted in Fig. 3(c), GSC consumes much less time and

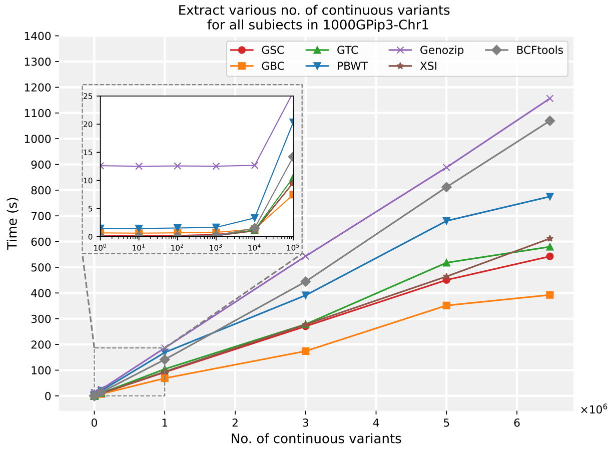

(a) Variant-based querying

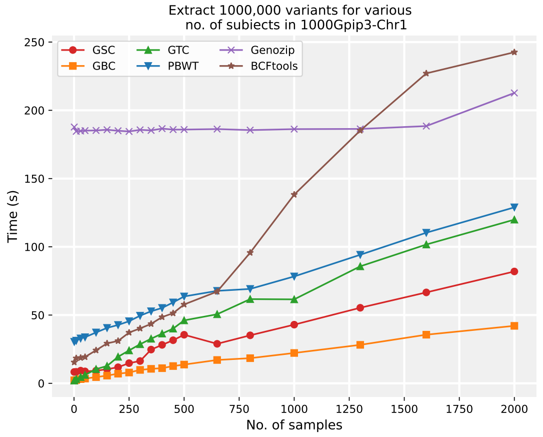

(b) Sample-based querying

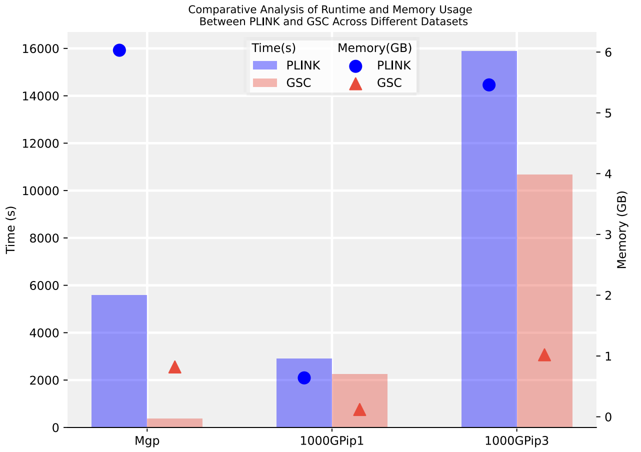

(c) Format conversion efficiency

Figure 3. Query and format conversion performance. (a) Performance of querying various no. of continuous variants for all samples in 1000GPip3-Chr1. (b) Performance of querying 1000,000 variants for various no. of samples in 1000GPip3-Chr1. (c) Performance comparison between PLINK and GSC in conversion to PLINK binary format.

memory space to convert the file format. Particularly in smaller sample datasets like Mgp, GSC is 15 times faster than PLINK. In large datasets from the 1000 Genomes Project, GSC still manages to attain a speedup of 30% with much smaller memory usage. The considerable reduction in conversion time and memory consumption highlights the benefit of saving VCF data with GSC compression, especially for the scenarios where PLINK is a downstream analysis option.

Discussion

In this article, by leveraging the sparse characteristics of preprocessed genotypes, we have crafted an efficient lossless compression algorithm namely GSC for VCF files, which supports fast genotype query. GSC attains competitive overall performance in terms of compression ratio, speed, memory usage, and query efficiency compared to other counterpart compressors. Specially, GSC shows superior compression ratios in both genotype compression and whole file compression. GSC also supports an efficient data conversion to PLINK binary format, which greatly facilitates the downstream analysis. For the sake of data management, GSC offers options to compress multiple VCF files into a single compressed archive (with the same sample count) and enables decompression of an archive into multiple VCF files according to the chromosomes. GSC also supports a streaming mode of operation that helps integrating GSC into pipelines. GSC can serve as a candidate efficient solution for VCF files storage and management.

Despite the promising performance of GSC, there are still some limitations. For example, currently GSC cannot handle VCF datasets containing no genotype information. This limitation is inherent to the design of GSC, which features a high degree of coupling between genotype information and the POS field. It deserves future work to improve the generalization ability by exploring new efficient methodologies to handle such datasets. Moreover, the block size in genotype data compression is fixed to the number of samples, which could maximize the redundancy reduction in the haplotype clustering and sparsification. Yet exploring the configurations with different block sizes could provide further insights into the scalability of GSC and more flexibility to the user.

Methods

The procedure of GSC is shown in a schematic diagram in Fig. 4. Given a VCF or BCF (binary version of VCF) file, GSC separates the annotation and genotype data, and compresses them with different strategies. Particularly, GSC leverages a hierarchical and block-based compression strategy to compress the genotype data. The genotype data is firstly divided into blocks each of which undergoes intra-block sorting, XOR processing, and sparse encoding. Afterward, the processed blocks are merged and encoded with general-purpose compressor BSC [28]. The data fields including CHROM, POS, ID, REF, ALT, QUAL, and FILTER are treated as fixed data streams. Each stream is partitioned into blocks of varying sizes, where each block's data volume is decided by the number of variant points in a genotypic data block. The stream blocks are also compressed with BSC. The remaining INFO and FORMAT data fields may contain subfields. Each subfield (except the genotype) is divided into fixed-size blocks and compressed independently. GSC not only supports lossless compression of VCF files but also facilitates rapid querying of genotype data. The key components of GSC are detailed as follows.

Preprocessing

The input VCF/BCF file firstly undergoes preprocessing to conform with the following compression. As illustrated in Fig. 5 (a), an input VCF/BCF file is likely composed of data from n chromosomes with each possessing v_i variants. Each variant, recorded in a line, contains h haplotypes denoted with '|' for phased and '/' for unphased alleles. Before compression, a variant containing multi-allelic is converted to multiple distinct variants where the first alternative

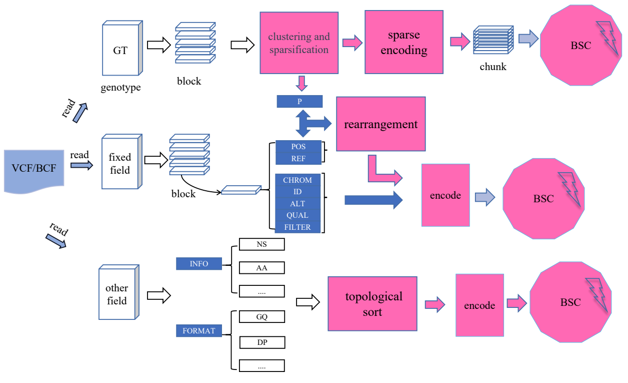

Figure 4. Overall workflow of GSC: In the initial step, VCF or BCF files are meticulously partitioned into multiple regions. Subsequently, differentiated processing strategies are applied based on the characteristics of each region to optimize the data structure. In the final step, all data, having been optimized, are further compressed using the BSC compressor to achieve efficient data storage.

allele remains unchanged, while the subsequent ones are denoted by special markers. For instance, as shown in Fig. 5 (a), a variant at POS = 1110696 of alleles ‘G’ and ‘T’ is divided into two distinct variants, i.e., with the first marked as G, <N>, and the second as T, <M> in ALT field. The <N> marker is exclusively used for the first split variant. To maintain the order of the variants, an additional index is added to the front of REF value for the variants with identical POS values. For example, indexes ‘1’ and ‘2’ are added in REF filed of the variants at POS = 1110696 as shown in Fig. 5 (a).

To encode the genotype data, each variant is represented by two variant bit vectors of size h following [15] as shown in Fig. 5 (b), where the bits indicate the type of mutation , i.e., ‘00’ for reference allele (‘0’), ‘01’ for non-reference allele (‘1’), ‘11’ for other non-reference alleles (‘2’), and ‘10’ for unknown alleles (‘.’). As such, the genotype of each chromosome can be encoded with $2hv_i$ variant bits and the genotype data of the entire VCF/BCF file is encoded with a total of $2h\sum_{i=1}^{n} v_i$ variant bits.

Compression of genotype data

After preprocessing, the bit-vector-encoded genotype data is partitioned into blocks, with each block containing s consecutive variants, i.e., $2s$ variant bit vectors per block. If the number of haplotypes h is smaller than 2^{13}, s is set to h, otherwise, s is set to 2^{13}. Consequently, a complete block contains $2sh$ bits. A chromosome i is divided into $\lceil v_i/s \rceil$ blocks ($\lceil \cdot \rceil$ is the ceiling function), and the whole genotype data is segmented into $\sum_{i=1}^{n} \lceil v_i/s \rceil$ blocks. Note that the last block of each chromosome usually contains less than s variants. The blocks can be processed in parallel to enhance the computational efficiency.

Each block of genotype data sequentially goes through haplotype clustering, sparsification, and sparse encoding to reach a compact representation. The details of the procedure are provided as follows:

- Haplotype clustering: as shown in Fig. 6 (a), the haplotypes (columns) within a block are clustered following [15] such that similar columns in terms of Hamming distance are grouped together. The new permutation order of the haplotypes is recorded in an array P.
- Sparsification: after the haplotype clustering, every consecutive eight columns in a block are considered as a group for sparsification, since a byte is the minimum unit of data storage, as shown in Fig. 6 (b). The total Hamming distance between all adjacent columns in a block can be calculated via $D = \sum_{i=1}^{h} d_i$, where d_i represents the Hamming distance between columns i and $i-1$ if $i \bmod 8 \not\equiv 1$, otherwise d_i is the Hamming weight of column i (the number of ones in column i). The sparsity of a block can be evaluated with the number of ones $\mho$ in the block. If $\mho > D$, the block is sparsified as follows. Within each column group, if the Hamming distance between a column X_i and its predecessor X_{i-1} ($i = 2, 3, ..., 8$) is less than the Hamming weight of X_i, X_i is replaced by $X_i^{'} = X_i \oplus X_{i-1}$, where $\oplus$ is an XOR operator. Note that the first column, i.e., X_1, in each group remains unchanged. Through the above transformation, the sparsity of a group can be reduced as the Hamming weight of $X_i^{'}$ is not greater than that of X_i.
- Sparse encoding: after sparsification, there might be a high prevalence of all-zero or duplicate bit vectors. The indexes of the all-zero and duplicate bit vectors in a block are recorded in binary vectors V_{zero}^{i} and V_{copy}^{i}, respectively, with the corresponding bits set to ‘1’, as shown in Fig. 6 (c). For V_{copy}^{i}, the corresponding indexes of the original copies are stored in another integer vector $A_{\text{origin\_pos}}^{i}$. Once the positions are properly recorded, the all-zero and duplicate bit vectors are removed from the block. The remaining block becomes sparse and the indexes of bits ‘1’ in each row are stored in an integer vector C_{index}^{i} where ‘0’ is defined as the delimiter of rows as shown in Fig. 6 (c). The vector C_{index}^{i} is further encoded into $C_{\text{index\_byte}}^{i}$ with delta coding and variable-length codes.

Due to the haplotype clustering, the haplotypes are repositioned and the original order must be recorded in the array P to ensure a lossless reconstruction of the data during the decompression. Nevertheless, if a genotype block contains h variants and the POS values are arranged in orderly as shown in Fig. 7, P can be omitted subject to a corresponding rearrangement of POS and REF values. As illustrated in Fig. 7, given P, we can permutate the POS and REF values accordingly such that the information of P is encoded in the rearranged POS and REF values. To recover P, we can simply sort the rearranged POS values back to the original order and record the permutation. Note that we could also apply delta encoding to the POS values and store the array P as it is, yet the space reduction in delta encoding of POS values cannot counteract the extra space required to store P. In the last genotype block, where the number of variants is usually not equal to h, P is plainly stored with variable byte encoding and the corresponding POS values are stored with delta encoding.

To improve the compression ratio while also maintain query speed, the genotype blocks are further merged into chunks. We adopt a chunk size of $l = 65536$ variants, i.e., each chunk consists of $m = \lfloor l/s \rfloor$ blocks ($\lfloor \cdot \rfloor$ is the floor function). The data of a single chromosome i is divided into $\lceil \lceil v_i/s \rceil / m \rceil$ chunks, and the entire genotype data is finally packed into $\sum_{i=1}^{n} \lceil \lceil v_i/s \rceil / m \rceil$ chunks. The chunks are compressed with the general-purpose compressor BSC.

Compression of other data fields

The INFO and FORMAT fields encompass a variety of subfields of phasing information for genotypes. Each subfield, along with the phasing data (except for the genotype itself) is divided into blocks of 8 MB and compressed using the BSC algorithm.

In a VCF/BCF file, determining the actual order of subfields is challenging when their order specified in the metadata section does not match their actual occurrence in the variant rows. To address this discrepancy, as the example shown in Fig. 8(a), we employ the HTSlib library [29] to parse the metadata and systematically extract the IDs for the INFO and FORMAT subfields. The IDs are then methodically cataloged in a ‘keys’ table, which includes the ‘Field’, ‘ID’, and their corresponding ‘key_id’ obtained by HTSlib. Based on the ‘keys’ table, the IDs of the INFO and FORMAT subfields in each variant can be mapped to a string of ‘key_id’, as shown in Fig. 8(b). We then introduce a Directed Acyclic Graph (DAG) to record the ‘key_id’ strings. As shown in Fig. 8(c), an initial DAG is constructed

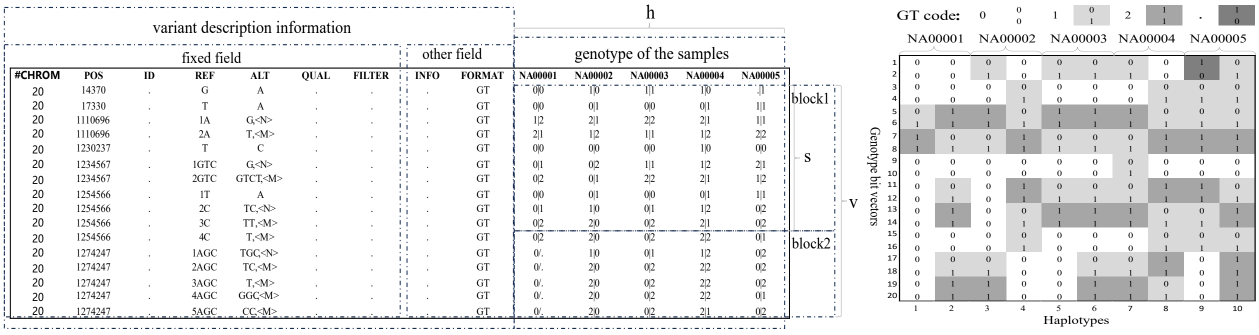

(a) Partitioning of VCF Files

(b) Genotypes encoding of in block 1

Figure 5. Preprocessing the input VCF file. (a) Splitting of specific variant rows and partitioning of VCF data for management. (b) Each genotype is encoded into two bits.

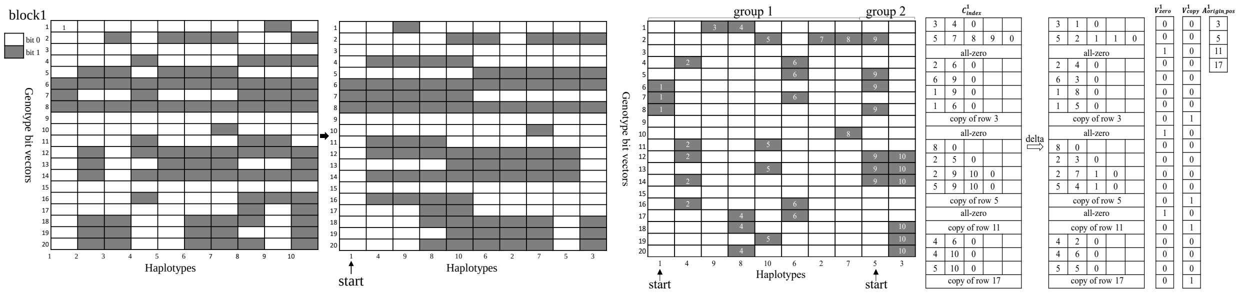

(a) Permutation of haplotypes in block 1

(b) Sparsificaition of block 1

(c) Encoding of haplotypes in block 1

Figure 6. The processing of a genotype data block. (a) Clustering the bit vector blocks: employing a nearest neighbor algorithm based on Hamming distance for sorting (b) Sparsificaition: Perform XOR operations on each column of bit data. (c) Sparse encoding: all-zero and copy bit vectors within the block are removed and marked, followed by documenting the specific positions of ’1’ in the remaining bit vectors of the block.

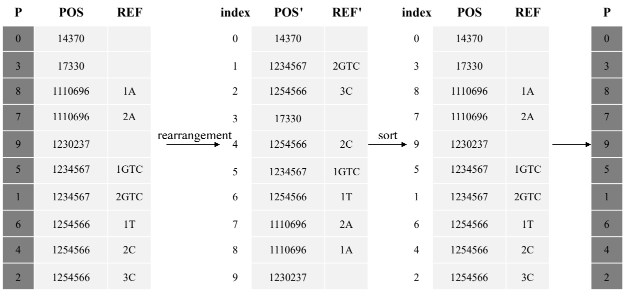

Figure 7. Rearrangement method used to map the array P to the POS and REF fields. During compression, the POS and REF fields are rearranged according to P. In the recovery phase, the indices are sorted according to the POS values to retrieve P.

with the first ‘key_id’ string recorded in Fig. 8(b). Afterward, the DAG is incrementally expanded with next ‘key_id’ string. This process is repeated until all ‘key_id’ strings are incorporated into the DAG. The final DAG is stored with a map data structure and the original ‘keys’ table can be retrieved with a topological sort of the final DAG. As such, lossless decompression of the INFO and FORMAT fields in the variant data is guaranteed. The field values are organized into separate data streams according to their field type. Each data stream is then divided into blocks of size 8MB to undergo BSC compression.

Decompression and Query

Downstream applications of VCF files are primarily focused on genotype analysis with analytical tools like VCFtools, BCFtools, and PLINK. Most existing VCF compression tools were designed to support only VCF and BCF formats. GSC implements lossless compression and two modes of decompression i.e., lossless and lossy modes. In lossless mode, GSC recovers the original file, whereas in the lossy mode it retains only the fixed data fields and the genotype data. Both modes enable the decompression of VCF/BCF formats, whereas the lossy mode also supports PLINK binary format.

The PLINK binary format (i.e., ‘bed’ format) does not include multi-allelic genotypes. Particularly, genotypes are represented as homozygous (0|0 and 1|1), heterozygous (1/0), and missing genotypes (0/1), where ‘0’ denotes a minor allele and ‘1’ a major allele. However, in VCF/BCF files, after preprocessing for multi-allelic genotypes, genotypes are denoted as homozygous (0|0 and 1|1), heterozygous (1/0 and 0/1), and missing genotypes (including ‘.’), with ‘0’ indicating a major allele and ‘1’ a minor allele. To convert the data into ‘bed’ format, we record the second type of non-reference allele ‘2’ back to ‘0’, and construct a mapping table based on the genotype variations (as shown in Fig. 9).

Random access of variants and/or samples is supported by GSC in decompression with specified conditions, including decompression mode, chromosome ID, position range within the chromosome, sample(s), ID of the variant, range of quality values, the minimum/maximum count/frequency of alternate allele among selected samples, and the maximum number of variant sites to decompress. GSC offers options for both variant-based and sample-based queries.

In variant-based query, given the queried variant(s), the corresponding chunks, blocks, and records are identified and decompressed. The chunks, blocks, and records are indexed with a B-tree like data structure in GSC, such that they could be quickly located. A variant is represented with a two-bit vector in GSC, which could be a regular vector, empty vector (all zeros), or a duplicated vector. To decompress a regular vector, a decoding procedure is conducted as a reverse of the encoding procedure described in Section *Compression of genotype data*. An empty vector is directly decompressed

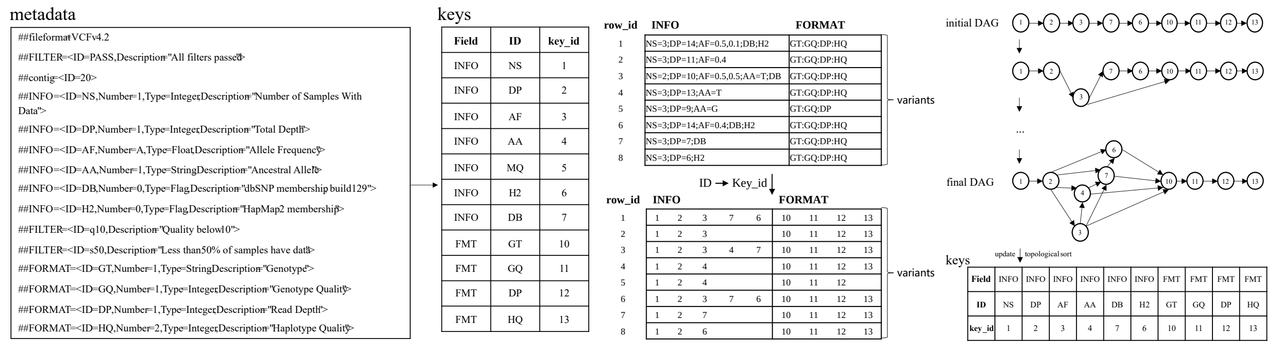

(a) Initializing the Mapping Table **(b)** Field ID Mapping **(c)** Retrieving the Original Field Order

Figure 8. Obtaining the true order of INFO and FORMAT subfield IDs. (a) Constructing a 'keys' lookup table. (b) Mapping the IDs of each variant's INFO and FORMAT fields to their corresponding key_ids. For example, for the variant line 'row_id=1', where the IDs in the INFO field is "NS", "DP", "AF", "DB", and "H2", the mapped sequence of 'key_id' is "1,2,3,7,6". (c) Constructing a Directed Acyclic Graph (DAG) based on the order of 'key_id'.

as all zeros. For duplicated vector, the original copy is identified and recovered according to V^i_{copy} and $A^i_{\text{origin\_pos}}$. Finally, the permutation order recorded in P and a byte-level lookup table are used to precisely locate the genotype and position of each variant within the query range.

In sample-based query, if the range of variants is specified, a similar procedure to variant-base query is performed to located the corresponding chunks, blocks, and records. The difference is that only the haplotypes of the queried samples are decompressed. We first determine the position(s) of the byte(s) in the bit vector(s) that encode the haplotypes based on the queried sample names and P, and then decompress the corresponding bytes to obtain the queried data.

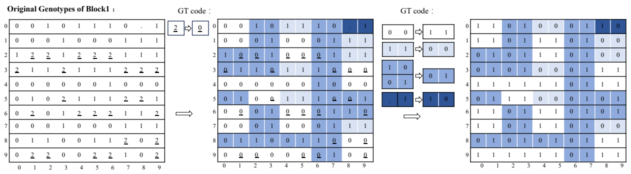

Figure 9. Conversion of genotype data of GSC to 'bed' Format.

Availability of Source Code and Requirements

Project name: GSC
Project home page: https://github.com/luo-xiaolong/GSC
Operating system(s): Linux
Programming language: C++
Other requirements: C++ compiler (e.g., g++)
License: GNU GPL
biotoolsID: gsc_genotype_sparse_compression
RRID: SCR_025071

Additional Files

Supplementary data Section 1. Information of the Compression Tools
Supplementary data Section 2. Datasets
Supplementary data Section 3. Experimental Environment
Supplementary data Section 4. Additional Results

Abbreviations

GSC: Genotype Sparse Compression; WGS: whole-genome sequencing; PBWT: positional burrows–wheeler transform; GT: Genotype; DAG: Directed Acyclic Graph; CR: Compression Ratio; CS: Compression Speed; DS: Decompression Speed; CMUE: Compression Memory Usage Effectiveness; DMUE: Decompression Memory Usage Effectiveness; KB: Kilobyte; XOR: Exclusive Or;

Authors' Contributions

X.L.L and Z.X.Z conceived and designed the GSC compression algorithm. Z.X.Z and Y.Z led the project's implementation. X.L.L was responsible for software development and performance optimization. X.L.L and Y.X.C conducted data analysis and benchmark tests. Y.X.C provided datasets and assisted in analyzing compression efficiency. X.L.L, Z.X.Z, and Y.X.C co-wrote the manuscript. All authors participated in research discussions and collectively decided on the content of the final version.

Competing Interests

The authors declare that they have no competing interests.

Data Availability

The data sets used in this study are publicly available from the following repositories:

- The *Mouse Genomes Project* data sets, including SNP and indel information across various mouse strains, were downloaded from database [30].

- The *1000 Genome Project — Phase 1* data sets, featuring integrated call sets of the first phase, were obtained from database [31].

- The *1000 Genome Project — Phase 3* data sets, encompassing the comprehensive release of phase 3 data, were accessed via database [32].

- The *kgenome* data sets, a consolidated file merging VCF data from chromosomes chr1 through chrX, totaling 23 chromosomes, were downloaded from database [33].

Note: The 'kgenome.vcf.gz' file represents an integration effort to combine VCF files for easier access and analysis.

Please refer to the respective repositories and documentation for detailed information on data usage permissions and restrictions.

Funding

This work was supported in part by the National Key Research and Development Program of China, under Grant 2022YFF1202104, in part by the National Natural Science Foundation of China, under Grant 61871272, and in part by the Guangdong Bigdata Engineering Technology Research Center for Life Sciences.

Acknowledgments

Not applicable

References

1. Danecek P, Auton A, Abecasis G, Albers CA, Banks E, DePristo MA, et al. The variant call format and VCFtools. Bioinformatics 2011;27(15):2156–2158.
2. Consortium GP, et al. A global reference for human genetic variation. Nature 2015;526(7571):68.
3. Halldorsson BV, Eggertsson HP, Moore KH, Hauswedell H, Eiriksson O, Ulfarsson MO, et al. The sequences of 150,119 genomes in the UK Biobank. Nature 2022;607(7920):732–740.
4. Danecek P, Bonfield JK, Liddle J, Marshall J, Ohan V, Pollard MO, et al. Twelve years of SAMtools and BCFtools. Gigascience 2021;10(2):giab008.
5. Deorowicz S, Danek A. GTShark: genotype compression in large projects. Bioinformatics 2019;35(22):4791–4793.
6. LeFaive J, Smith AV, Kang HM, Abecasis G. Sparse allele vectors and the savvy software suite. Bioinformatics 2021;37(22):4248–4250.
7. Durbin R. Efficient haplotype matching and storage using the positional Burrows–Wheeler transform (PBWT). Bioinformatics 2014;30(9):1266–1272.
8. Deorowicz S, Danek A, Kokot M. VCFShark: how to squeeze a VCF file. Bioinformatics 2021;37(19):3358–3360.
9. Lan D, Tobler R, Souilmi Y, Llamas B. genozip: a fast and efficient compression tool for VCF files. Bioinformatics 2020;36(13):4091–4092.
10. Layer RM, Kindlon N, Karczewski KJ, Consortium EA, Quinlan AR. Efficient genotype compression and analysis of large genetic-variation data sets. Nature Methods 2016;13(1):63–65.
11. Li H. BGT: efficient and flexible genotype query across many samples. Bioinformatics 2016;32(4):590–592.
12. Tatwawadi K, Hernaez M, Ochoa I, Weissman T. GTRAC: fast retrieval from compressed collections of genomic variants. Bioinformatics 2016;32(17):i479–i486.
13. Zheng X, Gogarten SM, Lawrence M, Stilp A, Conomos MP, Weir BS, et al. SeqArray—a storage-efficient high-performance data format for WGS variant calls. Bioinformatics 2017;33(15):2251–2257.
14. Salomon D, Motta G. Handbook of data compression. Springer Science & Business Media; 2010.
15. Danek A, Deorowicz S. GTC: how to maintain huge genotype collections in a compressed form. Bioinformatics 2018;34(11):1834–1840.
16. Wertenbroek R, Rubinacci S, Xenarios I, Thoma Y, Delaneau O. XSI—a genotype compression tool for compressive genomics in large biobanks. Bioinformatics 2022;38(15):3778–3784.
17. Zhang L, Yuan Y, Peng W, Tang B, Li MJ, Gui H, et al. GBC: a parallel toolkit based on highly addressable byte-encoding blocks for extremely large-scale genotypes of species. Genome Biology 2023;24(1):1–22.
18. Adhisantoso YG, Voges J, Rohlfing C, Tunev V, Ohm JR, Ostermann J. GVC: efficient random access compression for gene sequence variations. BMC Bioinformatics 2023;24(1):1–13.
19. Recommendation I. Information technology–Coded representation of picture and audio information–Progressive bi-level image compression. T82 (JBIG);.
20. Chang CC, Chow CC, Tellier LC, Vattikuti S, Purcell SM, Lee JJ. Second-generation PLINK: rising to the challenge of larger and richer datasets. Gigascience 2015;4(1):s13742–015.
21. Sudmant PH, Rausch T, Gardner EJ, Handsaker RE, Abyzov A, Huddleston J, et al. An integrated map of structural variation in 2,504 human genomes. Nature 2015;526(7571):75–81.
22. Adams DJ, Doran AG, Lilue J, Keane TM. The Mouse Genomes Project: a repository of inbred laboratory mouse strain genomes. Mammalian Genome 2015;26:403–412.
23. gzip;. https://www.gnu.org/software/gzip/. Accessed 16 May 2024.
24. zstd;. https://github.com/facebook/zstd.Accessed 16 May 2024.
25. Brotli;. Accessed 16 May 2024. https://github.com/google/brotli.
26. lz4;. https://github.com/lz4/lz4.Accessed 16 May 2024.
27. 7-zip;. https://7-zip.org/sdk.html.Accessed 16 May 2024.
28. BSC;. http://libbsc.com/. Accessed 16 May 2024.
29. Bonfield JK, Marshall J, Danecek P, Li H, Ohan V, Whitwham A, et al. HTSlib: C library for reading/writing high-throughput sequencing data. Gigascience 2021;10(2):giab007.
30. Mouse Genomes Project data sets;. ftp://ftp-mouse.sanger.ac.uk/REL-1807-SNPs_Indels/mgp.v6.merged.norm.snp.indels.sfiltered.vcf.gz. Accessed 16 May 2024.
31. 1000 Genome Project — Phase 1 data sets;. ftp://ftp.1000genomes.ebi.ac.uk/vol1/ftp/phase1/analysis_results/integrated_call_sets/.Accessed 16 May 2024.
32. 1000 Genome Project — Phase 3 data sets;. ftp://ftp.1000genomes.ebi.ac.uk/vol1/ftp/release/20130502/. Accessed 16 May 2024.
33. kgenome data sets;. https://ftp.cngb.org/pub/CNSA/data2/CNP0000702/data/VCF/kgenome.vcf.gz.Accessed 16 May 2024.

Click here to access/download
Supplementary Material
Supplementary data.docx

Click here to access/download
Supplementary Material
GSC_Evaluation_1.xlsx

Click here to access/download
Supplementary Material
GSC_Evaluation_2.xlsx
