# Supplementary material for: GSC: efficient lossless compression of VCF files with fast query
Source: Gigascience. 2024 Jul 19;13:giae046. doi: 10.1093/gigascience/giae046 (PMC11258903; doi:10.1093/gigascience/giae046)
Supplement: giae046_Supplemental_File [file giae046_supplemental_file.docx]

**Supplementary Materials**

**Contents**

[**1** **Information of the Compression Tools** 2](#_Toc168928748)

[**1.1** **Basic Information** 2](#_Toc168928749)

[**1.2** **Running Commands of the Tools** 2](#_Toc168928750)

[**2** **Datasets** 6](#_Toc168928751)

[**2.1** **Mouse Genomes Project** 6](#_Toc168928752)

[**2.2** **The 1000 Genomes Project — Phase 1** 6](#_Toc168928753)

[**2.3** **The 1000 Genomes Project — Phase 3** 6](#_Toc168928754)

[**3** **Experimental Environment** 7](#_Toc168928755)

[**4** **Additional Results** 7](#_Toc168928756)

1. **Information of the Compression Tools**
   1. **Basic Information**

The following compression tools were used in the experimental studies.

- PBWT v.3.0-8c25e5c (<https://github.com/richarddurbin/pbwt>)
- GTC v.1.1 (<https://github.com/refresh-bio/GTC>)
- GBC v.1.2 [(https://github.com/Zhangliubin/gbc)](file:///C:\Users\konea\Downloads\(https:\github.com\Zhangliubin\gbc))
- BCFtools v.1.11 (<https://github.com/samtools/bcftools/releases/download/1.11/bcftools-1.11.tar.bz2>)
- gzip v.1.6 (https://www.gnu.org/software/gzip/)
- Genozip v14.0.16 (https://github.com/divonlan/genozip)
- VCFShark v.1.1 (<https://github.com/refresh-bio/vcfshark>)
- XSI v.1.0(https://github.com/rwk-unil/xSqueezeIt)
- zstd v1.5.6(https://github.com/facebook/zstd)
- GSC v.1.0 (<https://github.com/luo-xiaolong/GSC>)
  1. **Running Commands of the Tools**

This section summarizes the running commands of the tools involved in the experiments. The contents wrapped in a pair of US-dollar symbols (i.e., $...$) are the parameters that the user needs to modify it. For example, $inputFile$ needs to be replaced with the correct vcf or vcf.gz path. Some tools do not support output vcf.gz format. The runtime of all tools is obtained through the /usr/bin/time directive to obtain the exact runtime.

**PBWT**

- Comperssion

./pbwt -readVcfGT $inputFile$ -writeAll $outputFile$

- Decompression (vcf)

./pbwt -readAll $inputFile$ -writeVcf - > $outputFile$

- Decompression (bcf)

./pbwt -readAll $inputFile$ -writeBcf - > $outputFile$

- sample query
  ./pbwt -readAll $inputFile$ -selectSamples $sampleFile$ -writeVcf - > $outputFile$
- Range of continuous variants query

./pbwt -readAll $inputFile$ -subrange $start$ $end$ -writeVcf - > $outputFile$

**GTC**

- Comperssion

./gtc compress -o $outputFile$ $inputFile$

- Decompression (vcf)

./gtc view -o $outputFile$ $inputFile$

- Decompression (bcf)

./gtc view -b -o $outputFile$ $inputFile$

- sample query
  ./gtc view -s $sample1,sample2,...$ -o $outputFile$ $inputFile$
- Many samples query:

./gtc view -s @$ samplesFlieList $ -o $outputFile$ $inputFile$

- Range of continuous variants query

./gtc view -r $chrom$:$start$-$end$ -o $outputFile$ $inputFile$

- Range of allele frequency query

./gtc view -minAF $minAF$ -maxAF $maxAF$ -o $outputFile$ $inputFile$

**XSI:**

- Compression:

./xsqueezeit -c -f $inputFile$ -o $outputFile$

- Decompression:

./xsqueezeit -d -f $inputFile$ -Ov -o $outputFile$

sample query
./xsqueezeit -x -s $sample1,sample2,...$ -f $inputFile$ -Ov -o $outputFile$

- Many samples query:

./xsqueezeit -x -S $samplesFlieList$ -f $inputFile$ -Ov -o $outputFile$

- Range of continuous variants query

./xsqueezeit -x -r $chrom$:$start$-$end$ -f $inputFile$ -Ov -o $outputFile$

**GBC**

In the following commands, ‘GBC’ is short for the command ‘java -jar gbc.jar’.

- Comperssion

GBC build $inputFile$ -o $outputFile$

- Decompression (vcf)

GBC extract $inputFile$ -o $outputFile$

- sample query
  GBC extract $inputFile$ -o $outputFile$ --select $sample1,sample2,...$ or GBC extract $inputFile$ -o $outputFile $ --select @$ samplesFlieList $
- Range of continuous variants query

GBC extract $inputFile$ -o $outputFile$ --range $chrom$:$start$-$end$

- Range of allele frequency query

GBC extract $inputFile$ --output $outputFile$ -fAF $minAF$-$maxAF$

**BCFtools**

- Compression:

./bcftools view -Ob -l9 -o $outputFile$ $inputFile$

- Decompression:

./bcftools view -Ov -o $outputFile$ $inputFile$

**gzip**

- Compression:

gzip -9 $inputFile$

- Decompression:

gzip -d $inputFile$

**Genozip:**

- Compression:

./genozip --no-test --licfile genozip_license -@ 1 -o $outputFile$ $inputFile$

- Decompression:

./genounzip -@ 1 -d $inputFile$ -o $outputFile$ $inputFile$

**VCFshark:**

- Compression:

./vcfshark compress -t 1 $inputFile$ $outputFile$

- Decompression:

./vcfshark decompress -t 1 $inputFile$ $outputFile$

**zstd:**

- Compression:

zstd $inputFile$

- Decompression:

zstd -d $inputFile$

**GSC**

Mode 1: Commands for Lossly Compression:

- Compression:

./gsc compress -M -o $outputFile$ $inputFile$

- Decompression(vcf):

./gsc decompress -M -o $outputFile$ $inputFile$

- Decompression(bcf):

./gsc decompress -M -b -l 0 -o $outputFile$ $inputFile$

Mode 2: Commands for Lossless Compression:

- Compression:

./gsc compress -o $outputFile$ $inputFile$

- Decompression:

./gsc decompress -o $outputFile$ $inputFile$

./gsc decompress -b -l 0 -o $outputFile$ $inputFile$

Mode 3: Commands for Query:

- sample query
  ./gsc decompress -M -s $sample$ -o $outputFile$ $inputFile$
- Many samples query

./gsc decompress -M -s @$ samplesFlieList $ -o $outputFile$ $inputFile$

- Range of continuous variants query

./ gsc decompress -M -r $chrom$:$start$-$end$ -s @$samplesFlieList$ -o $outputFile$ $inputFile$

- Many samples query for given range of variants

./ gsc decompress -M -r $chrom$:$start$-$end$ -o $outputFile$ $inputFile$

- Range of allele frequency query

./ gsc decompress -M --minAF $minAF$ --maxAF $maxAF$ -o $outputFile$ $inputFile$

Mode 4: Other Commands:

- Compress multiple files

./gsc compress --merge $inputFile1, inputFile2….. inputFileN$ -o $outputFile$

or

./gsc compress --merge @$inputFilesList$ -o $outputFile$

- Splitting files by chromosome during decompression:

./gsc decompress –split -o $outputFile$ $inputFile$

1. **Datasets**
   1. **Mouse Genomes Project**

**Description**

The dataset describes a total of 90,310,977 variant sites at 48 Mus musculus individuals. It consists of 1 VCF file for 22 chromosomes.

Size (vcf) : 182.22 GB

**Source**

The Mouse Genomes Project data sets were downloaded from:

https://ftp.cngb.org/pub/CNSA/data2/CNP0000702/data/VCF/mgp.v6.merged.norm.snp.indels.sfiltered.vcf.gz

- 1. **The 1000 Genomes Project — Phase 1**

**Description**

The dataset describes a total of 39,707,426 variant sites at 1, 092 H. sapiens individuals. It consists of 23 VCF files, corresponding to 22 autosomes, chromosomes X

Size (vcf) : 878.40 GB

**Source**

The 1000 Genome Project data sets were downloaded from:

<ftp://ftp.1000genomes.ebi.ac.uk/vol1/ftp/phase1/analysis_results/integrated_call_sets/>

- 1. **The 1000 Genomes Project — Phase 3**

**Description**

The dataset describes a total of 84,740,066 variant sites at 2, 504 H. sapiens individuals. It consists of 23 VCF files, corresponding to 22 autosomes, chromosomes X.

Size (vcf) : 794.84 GB

**Source**

The 1000 Genome Project data sets were downloaded from:

<ftp://ftp.1000genomes.ebi.ac.uk/vol1/ftp/release/20130502/>

Note: The file "kgenome.vcf" is a merger of VCF files from chromosomes chr1 to chrX, a total of 23 chromosomes, from the 1000 Genomes Project — Phase 3

<https://ftp.cngb.org/pub/CNSA/data2/CNP0000702/data/VCF/kgenome.vcf.gz>

1. **Experimental Environment**

The computer platform used in the experimental studies is configured with:

- OS: Ubuntu 18.04.2 LTS
- 2 Intel Xeon E5-2620 v3 CPUs, 6 double-threaded cores per CPU, summing up to a total of 24 threads, each clocked at 2.4 GHz,
- 128 GiB RAM.
- 1 SSDs of size 2 TiB, hdparm -t reported buffered read speed 456.04 MB/sec.
- Compiler: gcc v. 10.1.0 runing.

1. **Additional Results**

Additional results are provided in the Supplementary Worksheet ([GSC_Evaluation_1](file:///C:\Users\konea\Downloads\GSC_Evaluation_1.xlsx) and [GSC_Evaluation_2](file:///C:\Users\konea\Downloads\GSC_Evaluation_2.xlsx)).

**GSC_Evaluation_1. (Mgp, 1000GPip1 and 1000GPip3).**

**Description:** This worksheet includes the results of compressing genotype data using various tools and methods on the datasets Mgp, 1000GPip1, and 1000GPip3.

**GSC_Evaluation_2 (Lossess_compression_result) :**

**Description:** This worksheet presents the results of applying lossless compression algorithms to genotype data using various tools and methods on the datasets Mgp, 1000GPip1, and 1000GPip3.

**GSC_Evaluation_2 (Query_result):**

**Description:** This worksheet contains the performance results of executing queries on the compressed genotype data.

**GSC_Evaluation_2 (Format_Conversion_result):**

**Description:** This worksheet details the performance comparison of file format conversion between PLINK and GSC.

**GSC_Evaluation_2 (General_Compression_result):**

**Description:** This worksheet details the compression performance of GSC when using different general compression algorithms.
