# Supplementary material for: GSC: efficient lossless compression of VCF files with fast query
Source: Gigascience. 2024 Jul 19;13:giae046. doi: 10.1093/gigascience/giae046 (PMC11258903; doi:10.1093/gigascience/giae046)
Supplement: giae046_Response_to_Reviewer_Comments_Original_Submission [file giae046_response_to_reviewer_comments_original_submission.pdf]

Author's Response To Reviewer Comments

Dear Editor and Reviewers,

Thank you for the valuable comments which greatly improve the quality of our manuscript. According to the comments, we have correspondingly revised our manuscript and confirmed that all the comments are well handled. It is noted that the corresponding revisions in the manuscript are highlighted in blue font. The point-by-point response is provided as follows.

Editor:
Comment 1: Major comments of the reviewers include the need for more thorough and wider benchmarking and comparisons to state-of-the-art methods (reviewer #1) and the need to fix a number of problems when compiling and running the software (reviewer #2), among other concerns. In addition, please register any new software application in the bio.tools and SciCrunch.org databases to receive RRID (Research Resource Identification Initiative ID) and biotoolsID identifiers, and include these in your manuscript. Computational workflows should be registered in workflowhub.eu and the DOIs cited in the relevant places in the manuscript. These will facilitate tracking, reproducibility and re-use of your tool.

Response:
Many thanks for handling our manuscript. We have revised the manuscript and improved the experimental study according to the suggestions of the reviewers. The compiling and running issues of the software also have been fixed. We have registered our new software application in the bio.tools and SciCrunch.org databases. The RRID (SCR_025071) and biotoolsID (gsc_genotype_sparse_compression) identifiers of our tool have been provided in our manuscript. We also have made attempt to register the workflows in workflowhub.eu. Unfortunately, our application got stuck with unknown reason. We have contacted the administrator but currently there is no reply. We will cite the DOIs once we get through the registration.

Reviewer #1:
Minor comments:
Minor comment 1: - Table 4: The "original size" columns should not be sub-columns of the compression ratio columns (makes no semantic sense).

Response:
Thank you for pointing this out. We have revised the table to make "original size" a separate column.

Minor comment 2: Figure 3: It is unclear how the GT fields relate to the fixed fields. The information that fixed and other fields are divided into blocks is missing. Are the remaining fixed fields also sorted by P? Also, please clarify in the text how the permutation indices are mapped to the variants when the number of rows and columns in a block is different. Also, the POS field is originally sorted in ascending order, which can be efficiently encoded by first transforming it using delta encoding. Please clarify why the POS needs to be reordered first in this way and whether there is a performance penalty compared to storing the P as it is.

Response:
Many thanks for the suggestion. In the proposed GSC, only the POS and REF fields are reordered according to the permutation order of GT that is recorded in the array P, while the remaining fixed fields are unchanged. We have modified Figure 4 (previous Figure 3) to make this clear.

Regarding the storage of POS and P, there could be two solutions. In the first one, we could apply delta encoding to the POS field and store the array P as it is. Delta encoding is indeed an effective method to reduce the space of storing the POS field since it is sorted in ascending order. However, it requires extra space to store P. In the second solution, the POS field could be reordered according to P and only the reordered POS is stored, as P could be recovered from the reordered POS as shown in Figure 7. We have compared the two solutions in Section ‘Effects of the key components’, Page 3, and found the second one can achieve better compression ratio. The reason is that recording the reordered POS in the second

solution is equivalent to storing the array P in the first solution, yet the second one is exempt from storing the delta encoded POS.

In a block where the number of rows and columns is different (only occurring as the last block), the permutation indices in P are stored directly with variable-length byte encoding and the corresponding POS field is stored with delta encoding. We have added detailed description in the right column of Page 6.

Minor comment 3: - Figure 5a: The white right arrow is misplaced and barely visible.

Response:
Thank you for pointing this out. We have repositioned the arrow and enhanced its contrast for better visibility.

Minor comment 4: - Figure 5c: "encoding" -> "Encoding"

Response:
Thank you for pointing this out. We have fixed it in this revision.

Minor comment 5: - Figure 6: It should be made clear in the figure (caption) that the rearrangement sorts the rows by P and that the sort sorts the rows by POS.

Response:
Thank you for your suggestion. We have accordingly revised the figure (now Figure 7) caption to better explain the role of the arrays and the sequence of operations during the compression and recovery phases.

Minor comment 6: - Figure 7b: "FROMAT" -> "FORMAT"

Response:
Thank you for pointing this out. We have fixed the typo.

Minor comment 7: - Figure 7b: The order/arrangement of the tables is unclear.

Response:
Thank you for your suggestion. We have revised the figure caption to better describe how each table relates and interacts within the figure.

Minor comment 8: - Figure 7b: Please explain where and how the values of each field are stored.

Response:
Many thanks for the suggestion. Figure 8(b) (previous Figure 7b) is introduced to illustrate how IDs in INFO and FORMAT subfields transfer to key_id strings. The order of the IDs is stored in the DAG as shown in Figure 8(c). The field values are organized into separate data streams based on their field type. Each data stream is then divided into blocks, with each block being 8MB in size, to facilitate efficient storage. We have added description in the left column of Page 7.

Major comments:

Comment 1: - Please address the limitations of the proposed approach.

Response:
Thank you for your valuable suggestion. Despite the promising performance of GSC, there are some limitations of GSC. It cannot handle datasets containing no genotype information, such as the ExAC and gnomAD datasets. This limitation is inherent to the design of GSC, which features a high degree of coupling between genotype information and the POS fields, and is specifically optimized for the compression and retrieval of genotype data. It deserves future efforts to explore new methodologies and algorithms capable of incorporating and efficiently compressing these critical datasets. We have added discussion on the limitations and future work in the ‘Discussion’ section.

Comment 2: Please provide an evaluation of compression/decompression times and memory consumption, including information about multi-threading. This information is critical to understanding the scalability of the compared methods.

Response:
Thank you for the suggestion. We have provided the equivalent evaluation in our manuscript in Figure 1(b), where the metrics CS (Compression Speed) and DS (Decompression Speed) along with CMUE (Compression Memory Usage Efficiency) and DMUE (Decompression Memory Usage Efficiency) are represented in a radar chart. These indicators are designed to offer a comprehensive view of the performance and efficiency of each method under study. We also provide more detailed information of the test results in Section 4 of the Supplementary data. The experiments were conducted on a single processing thread. The configurations of the compared methods are provided in Section 3 of the Supplementary data. We have revised the description in the 'Compression performance' section to make this clear.

Comment 3: To better evaluate GSC's performance, you should evaluate GSC on larger and more recent datasets such as HRC, ExAC, and gnomAD, which may contain other fields.

Response:
Thank you very much for the valuable suggestion. The HRC dataset is managed under the stewardship of the Sanger Institute, which imposes strict access controls. Despite our efforts to obtain permission, we have not been granted the access to this dataset. The lack of access has unfortunately limited our evaluations on the HRC dataset.

GSC is oriented for conventional VCF files and it is specifically optimized for datasets containing genotype information, which is tightly integrated with POS fields to achieve high compression ratios and efficient data retrieval. Unfortunately, this design makes GSC unsuitable for compressing datasets without genotype data like ExAC and gnomAD. We have discussed this limitation in the 'Discussion' section. GSC could probably be modified to handle ExAC and gnomAD by sacrificing some compression performance, yet more efficient methods are more desirable in our future work.

Comment 4: Also, you should compare GSC with state-of-the-art methods such as VCFShark, GenoZip, and XSI for whole VCF file compression.

Response:
Many thanks for the suggestion. We have involved XSI in the comparison with GSC in the compression of genotype data (results shown in Table 1 and Figure 1), and VCFShark and GenoZip in the compression of whole VCF files (results shown in Table 2). The query performance of XSI and GenoZip is also reported in Figure 3.

GSC shown better overall compression and query performance than XSI. Since GSC has to maintain some extra space to support the fast query functionality, it is compression ratio on whole VCF file is slightly inferior to VCFShark and GenoZip. It might be inappropriate to directly compare GSC with fast random accessibility to VCFshark and Genozip, which are not intended to support fast random access. Yet we have provided discussion on the results in the left column of Page 3.

Comment 5:- Section "Introduction": You mention here that the genotype data may only constitute a small part of a VCF file, but later in the paper you elaborate that larger datasets (1000GPip3 and Kgenome) consist primarily of genotype data. Please clarify this.

Response:
Thank you for pointing this out. In the introduction, we aimed to highlight the diverse nature of VCF files, which can contain a wide range of data types beyond genotype information, such as annotations, metadata, and non-genotypic variant data. The intent was to emphasize that, while genotype data is a significant component, VCF files are not limited to this type of data alone and can encompass a broad spectrum of genomic information. To avoid causing potential confusion to the reader, we have revised the

description in the ‘Introduction’ Section as follows:

“Most of the above methods primarily focus on the compression of genotype data while disregarding the other annotation data present in VCF files. Given that genotype data might constitute only a portion of VCF files, such as in the first phase of the 1000 Genomes Project dataset, relying solely on genotype compression is insufficient to alleviate the storage and transmission pressures of large VCF files.”

Comment 6:- Section "Data Description": It is unclear to me what you mean by to "test the compatibility of GSC". What is the added value of the Kgenome dataset? Why should a method fail on concatenated VCF data, while it works on the individual VCF data?

Response:
Many thanks for the suggestions and apologies for not being able to make it clear in our manuscript.

Our primary goal in testing the compatibility of the GSC tool was to specifically evaluate whether the compressor can handle VCF files containing multiple chromosomes, which are increasingly prevalent in genomics research. As datasets grow in size and complexity, it is essential to ensure that GSC remains compatible with these more complex datasets.

The Kgenome dataset, unlike individual VCF files from the 1000 Genomes Project phase 3 (1000GPip3) that typically contain genomic data for a single chromosome, comprises merged VCF files for all 22 autosomes and the X chromosome. This integration significantly elevates the dataset's complexity and size, making it an ideal benchmark for testing the compression capabilities of GSC on concatenated datasets. It also allows us to evaluate GSC’s proficiency in handling complex, real-world genomic datasets.

Some compression tools, such as PBWT, are designed with the assumption that data follows the structure of single chromosomes. When faced with concatenated VCF files that include data from multiple chromosomes, these tools may struggle to manage the increased complexity and volume of data. This limitation underscores the importance of developing compression tools that can adapt to the characteristics of concatenated datasets.
We have provided revised description in the Section “Data Description” (Page 2) to clarify this point.

Comment 7:- Section "Findings"/Table 1: In my opinion, the compression ratio comparison is biased because the tools use different block sizes, which usually has a non-negligible impact on the compression ratios obtained. This may also be the reason why GSC largely outperforms GTC and other methods across all datasets. Please investigate and clarify this, e.g., by presenting results obtained using similar block sizes for all methods. In addition, it would be interesting and important to see at what block size GSC's performance saturates.

Response:
Thank you for the valuable suggestion. To avoid the potential biases introduced by using different block sizes, we have re-run the experiments with GSC, GBC, and GTC configured the same block size. The new results are updated to Table 1 and Figure 1. The other methods including BCFtools, XSI, and PBWT do not support custom block size. Their configurations were not changed.

Currently, the block size of GSC is determined based on the number of samples, which maximized the redundancy reduction in the haplotype clustering and sparsification. Nevertheless, exploring the performance of GSC with different block sizes could provide further insights into the scalability of GSC and more flexibility to the user. We have added discussions on this point in the ‘Discussion’ Section.

Reviewer #2:
Comment 1: p2: "All compressors were run with a minimum two threads" - It's not clear if all compressors were using the same number of threads, and why you mention the minimum instead of a maximum number of threads. I also think it would be informative to see single-threaded performance comparison.

Response:
Many thanks for the suggestion. We have re-run the experiments and all compressors were configured to

use a single thread. We have provided the following description in the Section "Findings/Compression performance" of the manuscript (Page 2):
“All compressors were run with a single thread on the same operating system. The detailed software and hardware configurations are provided in Section 3 of the Supplementary data.”

Comment 2: p4: "Afterward, the processed blocks are merged and encoded with general-purpose compressor BSC" - It is interesting what considerations determined the choice to use BSC, and if other compressors / libraries were considered. E.g., brotli, zstd, lz4, lzma might be valid choices for some of the tasks in this context. It may be good to explain the reasons for choosing BSC in the text.

Response:
Thank you very much for the suggestion. The selection of backend encoder is critical to the performance of GSC. To investigate the effects of using different compressors as suggested, we have evaluated GSC with different compressors including brotli, zstd, lz4, lzma, and BSC. The comparison results in terms of Compression Ratio, Compression and Decompression Speed, and Memory Use Efficiency in 1000GPip1, 1000GPip3, and Mgp datasets have been provided in Figure 2. brotli achieved the highest compression ratio but was slower in terms of compression speed. lz4 excelled in compression speed and memory efficiency at a cost of lower compression ratio. lzma, zstd, and BSC obtained the better overall performance than brotli and lz4. Among them, BSC achieved the best compromise in terms of all performance metrics, which justifies our selection of BCS as the backend compressor of GSC. We have provided the results and discussions of this comparison study in the ‘Effects of the key components’ Section.

Comment 3: I recommend adding the zstd compressor to the comparison, as a more relevant example of a modern general purpose compressor.

Response:
Thank you for the recommendation. We have included zstd in the comparison study and the results are presented in Tables 1 and 2 as well as Figure 1.

Comment 4: It's not clear if GSC supports a streaming mode of operation, i.e., reading from standard input and writing to standard output. If it's supported, it's better to mention it in both the text and the github page, with example command lines. If it's not supported, I recommend adding such support, as it will greatly help integrating GSC into pipelines.

Response:
Thank you very much for pointing this out. GSC indeed supports streaming mode from standard input to standard output. We have updated accordingly in both the text (the ‘Discussion’ Section) and the GitHub page, which now includes an example ‘./gsc compress -i sample-8.vcf > sample-8.vcf.gsc’ to guide the users on utilizing GSC within a streaming context.

Comment 5: Compiling GSC produces many warnings, which does not inspire confidence in code quality. Please consider fixing all warnings. (I used "g++ (GCC) 12.3.1 20230508 (Red Hat 12.3.1-1)" on Fedora 37).

Response:
Many thanks for pointing this out. We have resolved the issues by meticulously revising our code to ensure it adheres to the best practices and standards in C++ programming. These revisions were tested using the same version of GCC you mentioned (GCC 12.3.1 on Fedora 37) to ensure that no warnings are produced during compilation. The latest version of GSC has been updated on our GitHub page.

Comment 6: Command line example from github page: "gsc compress [out_file] [in_file]" - It's not a good idea to allow specifying the input and output files this way, as it looks there might be a risk of swapping their position around, accidentally overwriting the input file. Instead I recommend requiring "--in [in_file]" and "--out [out_file]", to make it clear which file is the input and which one is the output. It will also make it easier to read GSC command lines in pipelines.

Response:
Thank you very much for the constructive feedback regarding the syntax of the command line interface. Following your recommendation, we have updated the command line interface to require explicit flags for input and output files. The new syntax now uses "--in [in_file]" and "--out [out_file]" to clearly designate the input and output files, respectively. We have updated the documentation on our GitHub page to reflect these changes and provide clear guidance for our users.

Comment 7: After building GSC, at first I could not make it work for compression. "./gsc" shows the help message mentioning both "compress" and "decompress" modes. However, trying compression on some of my data with "./gsc compress sample-8.vcf.gsc sample-8.vcf" did nothing and somehow just shown a help message for the decompression command. Adding "--out" helped, i.e., this command worked: "./gsc compress --out sample-8.vcf.gsc sample-8.vcf". I suggest improving the github documentation to make it clear how to run the command.

Response:
Thank you for pointing this out. We have conducted a thorough review and testing of the command line interface, specifically focusing on the issues you encountered with the "compress" command. We have identified and corrected the issue that caused the incorrect display of help messages and potentially hindered the execution of compression commands.
We have also tested the compression functionality using the example file "sample-8.vcf" as you mentioned, and we can confirm that the issue has been resolved. To prevent any future confusion, we have updated the GitHub documentation to clearly illustrate the correct usage of the compression command, especially emphasizing the necessity of the "--out" flag for clarity. The updated documentation now includes more explicit instructions and examples to ensure that users can easily understand and correctly use GSC without encountering similar issues.
Comment 8: After compression completed, unexpectedly there was no output file "sample-8.vcf.gsc" produced. Instead there were two files named "sample-8.vcf.gsc.dbs" and "sample-8.vcf.gsc.gti". I have to say that this is an unfortunate choice. Multi-files archives complicate file and data management, and make it harder to integrate this compressor into pipelines. E.g., imagine a scenario of taking a vcf file, compressing it with gsc, and immediately streaming to a remote server. Or vice versa, streaming gsc-compressed data from remote machine, decompressing it on the fly, and immediately streaming the data to the next analysis step. These usages are not possible, or at least inconvenient, when a gsc archive consists of multiple files. I hope you can reconsider and switch to a single-file archive format.

Response:
Thank you very much for the suggestion. We have revised the code to generate a single-file archive, using only the `.gsc’ extension. The updated version with these modifications has been thoroughly tested to ensure that it maintains the same level of compression efficiency and integrity. We have also updated the GitHub documentation to reflect these changes and provide clear guidance on using the new single-file format.

Comment 9:. Decompressing my data back to vcf with the command "./gsc decompress --out sample-8.vcf sample-8.vcf.gsc" did not produce a file "sample-8.vcf", which I expected it to produce. Instead it produced a file "sample-8.vcf.vcf". Please avoid modifying user-supplied file names and use them exactly as they are.

Response:
Thank you for pointing out the issue. We apologize for the inconvenience caused by the unexpected modification of the output file name. We have identified an oversight in the decompression command logic that led to the addition of an extra file extension. We have corrected this issue to ensure that the tool uses the file names exactly as specified by the user without any alterations.

Comment 10: The decompression command mentioned above crashed with a message "Segmentation fault (core dumped)". It produced partial vcf output, but not the entire file. I uploaded my test data so you can use it for testing (zstd-compressed): https://biokirr.com/Shared-Data/GSC/ . Since this is the first dataset I tried, this leaves a bad impression. It should be obvious that a data compressor must be rock-solid and

reliable.

Response:
Apologies for the difficulties you encountered while using GSC. We greatly appreciate your effort in testing our software and sharing your feedbacks. We have resolved the issue and conducted extensive testing across various datasets, including the data you kindly uploaded, to ensure that no similar issues remain. We have also updated our GitHub documentation and implementation to reflect these changes and improvements. Your insights have been invaluable to enhancing the robustness of our tool, and we are truly grateful for your contribution.
