# Supplementary material for: GSC: efficient lossless compression of VCF files with fast query
Source: Gigascience. 2024 Jul 19;13:giae046. doi: 10.1093/gigascience/giae046 (PMC11258903; doi:10.1093/gigascience/giae046)
Supplement: giae046_Response_to_Reviewer_Comments_Revision_1 [file giae046_response_to_reviewer_comments_revision_1.pdf]

Author's Response To Reviewer Comments

Dear Editor and Reviewers,
Thank you for the valuable comments which greatly improve the quality of our manuscript. According to the comments, we have correspondingly revised our manuscript and confirmed that all the comments are well handled. The point-by-point response is provided as follows.

Editor:
Comment 1: Before we proceed, please include the following reference in the bibliography for your code in the software heritage archive:
[xx] Luo X, Chen Y, Liu L, Ding L et al. GSC - Genotype Sparse Compression (Version 1). [Computer software]. Software Heritage, 2024.
https://archive.softwareheritage.org/swh:1:snp:40316de76f5212af5196d29b506b30defa0ff95c;
origin=https://github.com/luo-xiaolong/GSC
Please cite this reference from your code availability section ("An archival copy of the code is available via the Software Heritage Archive[xx]"

Response:
Many thanks for handling our paper. We have cited the reference from our code code availability section as [30] in this revision.

Comment 2: Please also fix the links and other minor issues mentioned in the email from our data curators (see below).
1. Kgenome Dataset
In the original version of your manuscript, you refer to the following:
The file "kgenome.vcf" is a merger of VCF files from chromosomes chr1 to chrX, a total of 23 chromosomes, from the 1000 Genomes Project — Phase 3
https://ftp.cngb.org/pub/CNSA/data2/CNP0000702/data/VCF/kgenome.vcf.gz
However, in the updated version of your manuscript, you have omitted the link to the Kgenome dataset. Please provide details of how a researcher can access the Kgenome dataset, and add these details to the manuscript.
2. Broken Link
Please note that there is a broken link in the References and in the Supplementary File for this manuscript. I refer to the following link in Reference 30:
ftp://ftp-mouse.sanger.ac.uk/REL-1807-SNPs_Indels/mgp.v6.merged.norm.snp.indels.sfiltered.vcf.gz
This link is not recognised, and the correct link is below:
https://ftp.cngb.org/pub/CNSA/data2/CNP0000702/data/VCF/mgp.v6.merged.norm.snp.indels.sfiltered.vcf.gz
Please correct the link in the References and in the Supplementary File.

Response:
We have fixed the links in this updated version. The link of Kgenome Dataset is provided in reference [34] and cited in the text. The broken link in the supplementary file has also been corrected.

Comment 3: At this point, you can also remove any highlighting in color /tracking of changes that was made for the purpose of peer review.

Response:
The highlighting in color/tracing of changes have been removed.

Reviewer #1: My comments were adequately addressed.

Response:
Many thanks for putting efforts in reviewing our paper.

Reviewer #2: Thank you for improving both the tool and the manuscript based on my suggestions. I tried the

updated tool and this time it worked without issues. I particularly appreciate that you changed to a single-file archive format, and that you fixed all compiler warnings. I have no further suggestions and I recommend accepting this paper.

Response:
Thank you very much for your valuable suggestions and positive feedbacks
