# Supplementary material for: GSC: efficient lossless compression of VCF files with fast query
Source: Gigascience. 2024 Jul 19;13:giae046. doi: 10.1093/gigascience/giae046 (PMC11258903; doi:10.1093/gigascience/giae046)
Supplement: giae046_Response_to_Reviewer_Comments_Revision_2 [file giae046_response_to_reviewer_comments_revision_2.pdf]

Author's Response To Reviewer Comments

Comment 1: In the "code availability" section, you include a doi for workflowhub, which is great. However, please move this doi to the bibliography as a numbered item, and cite it by reference number from the availability section (we treat DOIs as citable objects).

Response: The doi has been moved to the bib and cited in the text.

Comment 2: Please add ORCIDs for all authors to the title page, where available (no need to worry about the format at this stage - just include the OPRCIDs following the author names.

Response: The ORCIDs of all authors have been provided in the title page.
