# Supplementary material for: GSC: efficient lossless compression of VCF files with fast query
Source: Gigascience. 2024 Jul 19;13:giae046. doi: 10.1093/gigascience/giae046 (PMC11258903; doi:10.1093/gigascience/giae046)
Supplement: giae046_Reviewer_1_Report_Original_Submission — Jan Voges -- 3/20/2024 Reviewed [file giae046_reviewer_1_report_original_submission.pdf]

Reviewer Report

Title: GSC: Efficient lossless compression of VCF files with fast query

Version: Original Submission **Date:** 3/20/2024

Reviewer name: Jan Voges

Reviewer Comments to Author:

The paper presents a compression method for VCF files, called GSC. This task is considered crucial as the volume of data is increasing due to the burst of high-throughput sequencing technologies. The authors report that GSC achieves state-of-the-art performance in terms of compression ratio and random-access speed.Overall, the paper adequately documents the methods and techniques used. Also, an implementation is provided, including detailed instruction for reproducing the presented results. The software is published under an open-source license and has been benchmarked against publicly available data, so in summary, it adheres to open data and reproducibility standards.Minor comments:- Table 4: The "original size" columns should not be sub-columns of the compression ratio columns (makes no semantic sense).- Figure 3: It is unclear how the GT fields relate to the fixed fields. The information that fixed and other fields are divided into blocks is missing. Are the remaining fixed fields also sorted by P? Also, please clarify in the text how the permutation indices are mapped to the variants when the number of rows and columns in a block is different. Also, the POS field is originally sorted in ascending order, which can be efficiently encoded by first transforming it using delta encoding. Please clarify why the POS needs to be reordered first in this way and whether there is a performance penalty compared to storing the P as it is.- Figure 5a: The white right arrow is misplaced and barely visible.- Figure 5c: "encoding" -> "Encoding"- Figure 6: It should be made clear in the figure (caption) that the rearrangement sorts the rows by P and that the sort sorts the rows by POS.- Figure 7b: "FROMAT" -> "FORMAT"- Figure 7b: The order/arrangement of the tables is unclear.- Figure 7b: Please explain where and how the values of each field are stored.Major comments:- Please address the limitations of the proposed approach.- Please provide an evaluation of compression/decompression times and memory consumption, including information about multi-threading. This information is critical to understanding the scalability of the compared methods.- To better evaluate GSC's performance, you should evaluate GSC on larger and more recent datasets such as HRC, ExAC, and gnomAD, which may contain other fields.- Also, you should compare GSC with state-of-the-art methods such as VCFShark, GenoZip, and XSI for whole VCF file compression.- Section "Introduction": You mention here that the genotype data may only constitute a small part of a VCF file, but later in the paper you elaborate that larger datasets (1000GPip3 and Kgenome) consist primarily of genotype data. Please clarify this.- Section "Data Description": It is unclear to me what you mean by to "test the compatibility of GSC". What is the added value of the Kgenome dataset? Why should a method fail on concatenated VCF data, while it works on the individual VCF data?- Section "Findings"/Table 1: In my opinion, the compression ratio comparison is biased because the tools use different block sizes, which usually has a non-negligible impact on the compression ratios obtained. This may also be the reason why GSC largely outperforms GTC and other methods across all datasets. Please investigate and clarify this, e.g., by presenting results obtained using

similar block sizes for all methods. In addition, it would be interesting and important to see at what block size GSC's performance saturates.

Level of Interest

Please indicate how interesting you found the manuscript: Choose an item.

Quality of Written English

Please indicate the quality of language in the manuscript: Choose an item.

Declaration of Competing Interests

Please complete a declaration of competing interests, considering the following questions:

- Have you in the past five years received reimbursements, fees, funding, or salary from an organisation that may in any way gain or lose financially from the publication of this manuscript, either now or in the future?
- Do you hold any stocks or shares in an organisation that may in any way gain or lose financially from the publication of this manuscript, either now or in the future?
- Do you hold or are you currently applying for any patents relating to the content of the manuscript?
- Have you received reimbursements, fees, funding, or salary from an organization that holds or has applied for patents relating to the content of the manuscript?
- Do you have any other financial competing interests?
- Do you have any non-financial competing interests in relation to this paper?

If you can answer no to all of the above, write 'I declare that I have no competing interests' below. If your reply is yes to any, please give details below.

I declare that I have no competing interests.

I agree to the open peer review policy of the journal. I understand that my name will be included on my report to the authors and, if the manuscript is accepted for publication, my named report including any attachments I upload will be posted on the website along with the authors' responses. I agree for my report to be made available under an Open Access Creative Commons CC-BY license (http://creativecommons.org/licenses/by/4.0/). I understand that any comments which I do not wish to be included in my named report can be included as confidential comments to the editors, which will not be published.

Choose an item.

To further support our reviewers, we have joined with Publons, where you can gain additional credit to further highlight your hard work (see: https://publons.com/journal/530/gigascience). On publication of this paper, your review will be automatically added to Publons, you can then choose whether or not to claim your Publons credit. I understand this statement.

Yes Choose an item.
