# Supplementary material for: GSC: efficient lossless compression of VCF files with fast query
Source: Gigascience. 2024 Jul 19;13:giae046. doi: 10.1093/gigascience/giae046 (PMC11258903; doi:10.1093/gigascience/giae046)
Supplement: giae046_Reviewer_2_Report_Original_Submission — Kirill Kryukov, Ph.D. -- 3/23/2024 Reviewed [file giae046_reviewer_2_report_original_submission.pdf]

Reviewer Report

Title: GSC: Efficient lossless compression of VCF files with fast query

Version: Original Submission **Date:** 3/23/2024

Reviewer name: Kirill Kryukov, Ph.D.

Reviewer Comments to Author:

Review of the manuscript titled "GSC: Efficient lossless compression of VCF files with fast queryGSC: Efficient lossless compression of VCF files with fast query".The authors describe GSC, a new method of compressing VCF files. GSC's performance numbers look promising, so I think this is a welcome development. Support for outputting PLINK's binary format is a nice touch that will help integrating it into pipelines. I think this is a promising work that will be helpful to managing large colections of VCF files. However I still have some concerns about both the tool and the paper:1. p2: "All compressors were run with a minimum two threads" - It's not clear if all compressors were using the same number of threads, and why you mention the minimum instead of a maximum number of threads. I also think it would be informative to see single-threaded performance comparison.2. p4: "Afterward, the processed blocks are merged and encoded with general-purpose compressor BSC" - It is interesting what considerations determined the choice to use BSC, and if other compressors / libraries were considered. E.g., brotli, zstd, lz4, lzma might be valid choices for some of the tasks in this context. It may be good to explain the reasons for choosing BSC in the text.3. I recommend adding the zstd compressor to the comparison, as a more relevant example of a modern general purpose compressor.4. It's not clear if GSC supports a streaming mode of operation, i.e., reading from standard input and writing to standard output. If it's supported, it's better to mention it in both the text and the github page, with example command lines. If it's not supported, I recommend adding such support, as it will greatly help integrating GSC into pipelines.5. Compiling GSC produces many warnings, which does not inspire confidence in code quality. Please consider fixing all warnings. (I used "g++ (GCC) 12.3.1 20230508 (Red Hat 12.3.1-1)" on Fedora 37).6. Command line example from github page: "gsc compress <options> [out_file] [in_file]" - It's not a good idea to allow specifying the input and output files this way, as it looks there might be a risk of swapping their position around, accidentally overwriting the input file. Instead I recommend requiring "--in [in_file]" and "--out [out_file]", to make it clear which file is the input and which one is the output. It will also make it easier to read GSC command lines in pipelines.7. After building GSC, at first I could not make it work for compression. "./gsc" shows the help message mentioning both "compress" and "decompress" modes. However, trying compression on some of my data with "./gsc compress sample-8.vcf.gsc sample-8.vcf" did nothing and somehow just shown a help message for the decompression command. Adding "--out" helped, i.e., this command worked: "./gsc compress --out sample-8.vcf.gsc sample-8.vcf". I suggest improving the github documentation to make it clear how to run the command.8. After compression completed, unexpectedly there was no output file "sample-8.vcf.gsc" produced. Instead there were two files named "sample-8.vcf.gsc.dbs" and "sample-8.vcf.gsc.gti". I have to say that this is an unfortunate choice. Multi-files archives complicate file and data management, and make it harder to integrate this compressor into pipelines. E.g., imagine a

scenario of taking a vcf file, compressing it with gsc, and immediately streaming to a remote server. Or vice versa, streaming gsc-compressed data from remote machine, decompressing it on the fly, and immediately streaming the data to the next analysis step. These usages are not possible, or at least inconvenient, when a gsc archive consists of multiple files. I hope you can reconsider and switch to a single-file archive format.9. Decompressing my data back to vcf with the command "./gsc decompress --out sample-8.vcf sample-8.vcf.gsc" did not produce a file "sample-8.vcf", which I expected it to produce. Instead it produced a file "sample-8.vcf.vcf". Please avoid modifying user-supplied file names and use them exactly as they are.10. The decompression command mentioned above crashed with a message "Segmentation fault (core dumped)". It produced partial vcf output, but not the entire file. I uploaded my test data so you can use it for testing (zstd-compressed): https://biokirr.com/Shared-Data/GSC/ . Since this is the first dataset I tried, this leaves a bad impression. It should be obvious that a data compressor must be rock-solid and reliable.

Level of Interest

Please indicate how interesting you found the manuscript: Choose an item.

Quality of Written English

Please indicate the quality of language in the manuscript: Choose an item.

Declaration of Competing Interests

Please complete a declaration of competing interests, considering the following questions:

- Have you in the past five years received reimbursements, fees, funding, or salary from an organisation that may in any way gain or lose financially from the publication of this manuscript, either now or in the future?
- Do you hold any stocks or shares in an organisation that may in any way gain or lose financially from the publication of this manuscript, either now or in the future?
- Do you hold or are you currently applying for any patents relating to the content of the manuscript?
- Have you received reimbursements, fees, funding, or salary from an organization that holds or has applied for patents relating to the content of the manuscript?
- Do you have any other financial competing interests?
- Do you have any non-financial competing interests in relation to this paper?

If you can answer no to all of the above, write 'I declare that I have no competing interests' below. If your reply is yes to any, please give details below.

I declare that I have no competing interests

I agree to the open peer review policy of the journal. I understand that my name will be included on my report to the authors and, if the manuscript is accepted for publication, my named report including any attachments I upload will be posted on the website along with the authors' responses. I agree for my report to be made available under an Open Access Creative Commons CC-BY license (http://creativecommons.org/licenses/by/4.0/). I understand that any comments which I do not wish to be included in my named report can be included as confidential comments to the editors, which will not be published.

Choose an item.

To further support our reviewers, we have joined with Publons, where you can gain additional credit to further highlight your hard work (see: https://publons.com/journal/530/gigascience). On publication of this paper, your review will be automatically added to Publons, you can then choose whether or not to claim your Publons credit. I understand this statement.

Yes Choose an item.
